# Effects of magnesium valproate adjuvant therapy on patients with dementia: A systematic review and meta-analysis

**DOI:** 10.1097/MD.0000000000029642

**Published:** 2022-08-05

**Authors:** ChenQi Zhang, LingQi Sun, HongBin Sun

**Affiliations:** a Department of Special-needed Medical, Chengdu BOE Hospital, Chengdu, Sichuan Province, China; b Department of Neurology, The Air Force Hospital of Western Theater Command, Chengdu, Sichuan Province, China; c Department of Neurology, Sichuan Academy of Medical Sciences and Sichuan, Provincial People’s Hospital, Chengdu, China.

**Keywords:** dementia, magnesium valproate, meta-analysis

## Abstract

**Background:**

Current research has found contradictory results on the treatment of magnesium valproate (VPM) in patients with dementia (PwD).

**Objectives:**

Here, we conducted a meta-analysis to evaluate the efficacy and safety of VPM in the adjuvant treatment of PwD.

**Purpose:**

Current research has found contradictory results on the treatment of VPM in PwD. Here, we conducted a meta-analysis to evaluate the efficacy and safety of VPM in the adjuvant treatment of PwD.

**Methods:**

MEDLINE via PubMed, Cochrane Library, EBSCO, Embase, China National Knowledge (CNKI), and Wan Fang databases were researched to gather relevant data on magnesium valproate assistant therapy for patients with dementia (PwD) by using medical subject headings and term words.

**Results:**

After the final screening, 22 RCT studies (a total of 1899 participants) were included in this meta-analysis, which compared VPM adjuvant treatment with antidementia or psychotropic drug monotherapy. Significant differences were found in the scores on mini-mental state examination (*P* = .028), Alzheimer disease assessment scale cognitive subscale (*P* < .05), Bech-Rafaelsen Mania Rating Scale (*P* < .05), behavioral pathology in Alzheimer disease rating scale (*P* = .001), activities of daily living (*P* < .05), and Pittsburgh Sleep Quality Index (*P* < .05). Besides, the levels of inflammatory factors including IL-1β, IL-6, and TNF-α were significantly lower than those in the monotherapy group (*P* < .05). While there was no increase in the incidence of adverse events (*P* = .383), VPM as an assistant therapy is generally well tolerated in PwD.

**Conclusion:**

By meta-analysis, evidence was found to support VPM additional used for the treatment of cognitive function, psychiatric symptoms, or disease improvement in PwD. VPM may be a potential drug to aid in the treatment of dementia patients. However, there was lack of enough evidence to classification of dementia severity in our inclusion study. More research is still needed, including clinical trials evaluating VPM as a complementary therapy.

## 1. Introduction

According to US population estimates of people with clinical AD and mild cognitive impairment, an estimated 6.2 million Americans aged 65 and older are living with AD today. By 2060, the number could grow to 13.8 million.^[1]^ Consistent with that we can learn from the 2020 report of the Lancet Commission, there are about 50 million people living with dementia worldwide, especially in low-income and middle-income countries, and that number is expected to rise to 152 million by 2050. Dementia affects individuals, their families, and the social economy, its costs estimated at US$1 trillion annually.^[2]^ In addition, the costs can also include an increased risk of emotional distress and negative physical and mental health outcomes for family caregivers.^[3]^ These figures reflect dementia patients have a higher burden of illness compared with other diseases.

However, there is no pharmacological treatment currently for dementia that can delay or stop the damage and destruction of neurons, which is the reason of Alzheimer symptoms and makes the disease fatal. The US Food and Drug Administration (FDA) has allowed 5 drugs for the treatment of AD until 2020: rivastigmine, galantamine, donepezil, memantine, and memantine combined with donepezil now, but none of these medicines are approved to treat behavioral and psychiatric symptoms of PwD.^[3]^ Meanwhile, evidence-based treatment guidelines for dementia suggest that anticonvulsants are not recommended in general, but state that in some patients, we could take them into consideration.^[4]^

Although not permitted, several drugs are still used in clinical practice for the treatment of PwD, one of the most commonly prescribed is the newer atypical antipsychotic agents; however, studies have shown that these drugs may increase the risk of stroke and death in PwD. Benzodiazepines, a common first-line treatment, have been associated with the risk of falls and disinhibition, which may increase agitation and aggression.^[5]^ In addition, there is currently little supportive data in clinical trials of antidepressant efficacy.^[6]^ These findings emphasize the urge to consider increasing more potential effective medicine in dementia-associated clinical trials.

Taking the pathophysiology of dementia into consideration, it included beta-amyloid and tau deposits along with inflammation and atrophy. Moreover, in animal studies, the accumulation of beta-amyloid can also cause seizures.^[7–9]^ By consulting the relevant literature, the use of antiepileptics agents (AEDs) in PwD has been supported by case reports and a modest amount of clinical research, especially carbamazepine and divalproex. Of these 2 agents, indeed, divalproex sodium offers the advantage of fewer drug interactions and adverse effects in this population.^[6]^

In clinical and laboratory studies, a decreased magnesium concentration was found in various tissues of PwD, including cerebral spinal fluid (CSF), red blood cells, plasma, and hair,^[10]^ reduced magnesium levels in the hippocampus particularly, seem to be an important factor in the pathogenesis of AD. There is new support for the neuroprotective effect of magnesium based on animal studies, suggesting that magnesium treatment at the early stage of dementia patients may delay their cognitive decline.^[11,12]^

According to the in vitro and in vivo studies, valproic acid may have neuroprotective effects on PwD, through a variety of potential mechanisms including actions on gamma-aminobutyric acid (GABA) and N-methyl-D-aspartate (NMDA) receptors, prevention of beta-amyloid aggregation, decreased beta-amyloid and neurotic plaque production, and induction of neurogenesis to ameliorate the symptoms of dementia.^[13]^

On the other hand, magnesium for its ability to affect vascular function in addition to neuronal function.^[14]^ Thus, based on these theories, VPM may be affecting cognitive function in multiple distinct ways.

As early as 2003, Lonergan et al first published a meta-analysis on the use of valproic acid (VPA) in the treatment of agitation of PwD, the results showed that low-dose VPA is ineffective in treating agitation among demented patients, and that high-dose divalproex sodium is associated with an unacceptable rate of adverse effects.^[14]^ Subsequently, Lonergan et al reached the same conclusion in a 2008 updated system evaluation.^[13]^ In addition, several trials have tested the antiagitation effect of VPA in PwD with negative results.^[15]^

Although most of the experiments showed negative results of valproic acid for dementia patients, most of these tests used sodium valproate as a single therapy to compare with the placebo group. In China, many controlled studies using magnesium valproate as adjuvant therapy showed positive effects on dementia patients. Hence, current research has found contradictory results on the treatment of magnesium valproate. It requires further investigation and standardized ways to evaluate the effects of magnesium valproate on cognitive function in dementia patients. Currently, there is a lack of meta-analysis focusing on cognitive improvement and disease-modifying VPM-assisted therapy in the current peer-reviewed literature. Thus, we aimed to likely analyze the efficacy and safety of VPM adjuvant therapy of PwD based on RCTs.

## 2. Methods

There are no real patients participating, so ethical approval in our trial is not required. Based on the Cochrane Review Methods, PRISMA,^[16]^ and MOOSE^[17]^ principles were employed to predesign search methods, selection and exclusion criteria, basic data extraction, literature quality evaluation, and final statistical analysis. The protocol of our trial was registered on the International Platform of Registered Systematic Review and Meta-analysis Protocols with the registration number of INPLASY2021110038 and the DOI number is 10.37766/inplasy2021.11.0038 (https://inplasy.com/inplasy-2021-11-0038/).

### 2.1. Search strategy

Online databases including the MEDLINE via PubMed (1972 to October 2021), Cochrane Library databases (2001 to October 2021), and EBSCO (1986 to October 2021), Embase (1982 to October 2021) were comprehensive searched by 2 searchers. We do not have restrictions on language, but the search object was restricted to humen. To obtain the search results, the search strategy was conducted by using medical subject headings (Mesh) and term words, such as “Valproate Magnesium” [Mesh], “Valproic acid magnesium,” “Magnesium dipropyl acetate,” “Dementia” [Mesh], “dement*,” “Alzheimer*,” “Huntington*,” and so on (see Supplementary Materials, Supplemental Digital Content, http://links.lww.com/MD/G935). Additionally, we carefully screened all references relevant to the included studies to avoid inappropriate omissions.

### 2.2. Study selection

Inclusion criteria for our clinical studies: (1) RCT, (2) VPM as an adjunctive therapy provided to the monotherapy group, and (3) the study provided detailed and clear outcome of interest. On the other hand, exclusion criteria include one of the following: (1) observational studies; (2) not about dementia; (3) not about oral VPM; (4) not report diagnostic criteria of dementia; (5) the outcome data is not clear; (5) studies in which VPM as a monotherapy and compared with a control group. According to the above criteria, we finally identified a total of 22 RCTs.

### 2.3. Data extraction

Two authors separately extracted relevant information, which is helpful for our study from each included trial, if there exist any discrepancies between the 2 authors, we resolved it by discussion or consulting with the senior reviewer. We established a data extraction form (Table 1), which included the study ID (combined author name with publication date), sample sizes, mean age, gender, details of participants, diagnostic criteria, treatment and control intervention, duration time, main outcome measures, and adverse events.

**Table 1:** Basic characteristics of all included studies.

Study ID	No. Randomed	Mean age (yr)	Gender (M:F)	Population (diagnosis)	(A) Treatment intervention	Duration time	Outcome	AE (%)
					(B) Control intervention			
Su^[18]^	84 (42:42)	(A) 74.26 ± 6.14	(A) 16:26	-Mild AD (Guideline)**-Barthel index**: (A) 82.16 ± 7.33(B) 80.75 ± 7.29**-MMSE**: (A) 20.94 ± 3.01(B) 20.53 ± 2.64	(B) +VPM 0.25 mg qd	1 m/2 m/3 m	**-MMSE**:(A) 23.57 ± 2.86/26.02 ± 3.07/27.56 ± 3(B) 22.36 ± 2.57/24.48 ± 2.82/25.12 ± 3.11	(A): 10 (23.81)(B): 12 (28.57)
	84 (42:42)	(B) 75.14 ± 6.05	(B) 19:23		Donepezil 5 mg qd, after 1 week of treatment, the dose was increased to 10 mg qd		**-NPI**: (A):28.54 ± 6.37/20.10 ± 5.33/14.56 ± 3.27 (B):31.56 ± 6.28/24.37 ± 5.69/17.33 ± 4.02	
Wang and Xue^[19]^	86 (43:43)	(A) 68.94 ± 4.43	(A) 24:19	-AD (Guideline)**-BEHAVE-AD** ≥ 8**-MMSE**:(A) 16.48 ± 6.03(B) 16.57 ± 5.86	(B) + VPM 0.25 mg qd, after 1 week of treatment, the dose was increased to 0.5 mg qd	1 m/3 m	**-MMSE**:(A):18.63 ± 4.25/22.01 ± 3.62 (B):17.93 ± 4.71/19.59 ± 3.96	(A):3 (6.98)(B):6 (13.95)
	86 (43:43)	(B) 69.80 ± 4.84	(B) 23:20		Donepezil 5 mg qd, after 1 week of treatment, the dose was increased to 10 mg qd		**-EHAVE-AD**:(A) 12.63 ± 3.74/6.01 ± 2.13(B):13.85 ± 3.96/9.95 ± 3.02	
							**-FIM**:(A) 78.05 ± 5.87/ 90.53 ± 4.11(B) 76.14 ± 5.61/ 83.47 ± 5.04	
Li and Zhan^[20]^	78 (39:39)	(A) 72.06 ± 4.57	(A) 23:16	AD (The 7th edition of the Neurology Book)**-MMSE**:(A) 18.41 ± 2.37(B) 18.29 ± 2.16	(B) + Magnesium valproate sustained-release tablets 250mg bid	3 m	**-MMSE:**(A) 21.57 ± 2.52 (B) 20.06 ± 2.35	(A):3 (7.69)(B):5 (12.82)
	78 (39:39)	(B) 71.58 ± 4.29	(B) 24:15		Donepezil 5 mg qd, after 1 week of treatment, the dose was increased to 10 mg qd		**-PSQI:**(A) 9.74 ± 1.38 (B) 14.62 ± 1.47	
							**-MOCA:**(A) 24.68 ± 3.14 B) 21.35 ± 2.91	
Zhu^[15]^	76 (38:38)	(A) 73.22 ± 5.23	(A) 27:11	AD (Guideline)**-MMSE**:(A) 11.24 ± 1.21(B) 11.57 ± 1.17	(B) + Magnesium valproate sustained-release tablets 0.25 mg qd, after 1 week of treatment, the dose was increased to 0.5 mg qd	3 m	**-MMSE**:(A) 21.36 ± 2.34 (B) 16.83 ± 2.12	(A): 6 (15.79)(B): 3 (7.89)
	76 (38:38)	(B) 75.18 ± 5.16	(B) 23:15		Galantamine, the initial dose was 4 mg bid, after 4 weeks of treatment, the dose was adjusted to 8 mg bid		**-ADL**:(A) 16.53 ± 1.35 (B) 24.58 ± 1.56	
							**-ADAS-cog**:(A) 15.48 ± 3.57 (B) 26.78 ± 3.32	
Zhang^[21]^	100 (50:50)	(A) 77.61 ± 8.15	(A) 25:25	AD (CCMD-3)	(B) + VPM 25 mg bid, after 1 week of treatment, the dose was increased to 50 mg bid	3 m	**-IL-1β (ng/L**):(A) 0.23 ± 0.11 (B) 0.41 ± 0.13	(A): 8 (14.00)(B): 3 (6.00)
	100 (50:50)	(B) 73.75 ± 6.32	(B) 24:26		Donepezil 5 mg qd, after 1 week of treatment, the dose was increased to 10 mg qd		**-IL-6 (ng/L**):(A) 84.02 ± 19.01 (B) 138.02 ± 38.01	
							**-TNF-α (μg/L**):(A) 107.01 ± 23.02 (B) 164.02 ± 25.01	
Wei^[22]^	94 (47:47)	(A) 61.57 ± 3.61	(A) 25:22	AD (CCMD-3)**-HAMD**:(A) 13.73 ± 1.88(B) 13.52 ± 1.72	(B) + Magnesium valproate sustained-release tablets 250 mg bid	3 m	**-PSQI**:(A) 7.33 ± 1.14 (B) 12.47 ± 1.27	NA
	94 (47:47)	(B) 61.85 ± 3.84	(B) 27:20		Donepezil 5 mg qd, after 1 week of treatment, the dose was increased to 10 mg qd		**-ADAS-cog**:(A) 24.57 ± 3.51 (B) 37.25 ± 3.84	
Wang^[23]^	90 (45:45)	(A) 74 ± 4	(A) 24:21	AD (CCMD-3)**-HAMD** ≤ 17**-MMSE**:(A) 12.1 ± 2.3(B) 12.2 ± 1.4	(B) + VPM 25 mg bid, after 1 week of treatment, the dose was adjusted to 50 mg bid	3 m	**-MMSE**:(A) 22.9 ± 4.3 (B) 16.5 ± 3.3	(A): 9 (20.00)(B): 7 (15.60)
							**-ADL**:(A) 16 ± 5 (B) 23 ± 6	
							**-ADAS-cog**:(A) 21.3 ± 4.2 (B) 27.3 ± 3.6	
	90 (45:45)	(B) 74 ± 4	(B) 26:19		Donepezil 5 mg qd, after 1 week of treatment, the dose was increased to 10 mg qd		**-IL-1β (ng/L**):(A) 0.25 ± 0.09 (B) 0.43 ± 0.11	
							**-IL-6 (ng/L**):(A) 84 ± 19 (B) 138 ± 38	
							**-TNF-α (μg/L**):(A) 107 ± 23 B) 164 ± 25	
Bi^[24]^	104 (52:52)	(A) 69.1 ± 2.2	(A) 31:21	AD (CCMD**-HAMD** ≤ 17**-HIS** < 4**-MMSE**:(A) 6.57 ± 1.40(B) 6.54 ± 1.39	(B) + Magnesium valproate sustained-release tablets 250 mg bid	4 w/8 w/16 w	**-MMSE**:(A) 10.04 ± 1.81/18.81 ± 2.05/25.39 ± 2.21 (B) 8.36 ± 2.27/12.53 ± 1.97/19.04 ± 4.68	(A):8 (15.4)(B):6 (11.5)
	104 (52:52)	(B) 68.2 ± 1.9	(B) 32:20		Donepezil 2.5–5 mg qd, after 1 week of treatment, the dose was increased to 5–10 mg qd		**-ADL**:(A) 47.24 ± 3.16/37.97 ± 3.04/24.48 ± 5.19(B) 49.97 ± 3.28/41.19 ± 2.56/35.16 ± 4.32	
Ao et al^[25]^	116 (58:58)	(A) 64.75 ± 10.36	(A) 33:25	Moderate AD (CCMD): **-MMSE**:(A) 23.8 ± 2.1(B) 23.9 ± 2.4	(B) + Magnesium valproate sustained-release tablets 250 mg qd, after 1 week of treatment, the dose was increased to 500 mg qd	16 w	**-MMSE**:(A) 21.5 ± 2.6 (B) 22.9 ± 2.2	(A):24 (41.4)(B):21 (36.2)
							**-ADL**:(A) 28.4 ± 4.4 (B) 26.5 ± 5.4	
							**-MoCA**:(A) 17.1 ± 2.1 (B) 19.3 ± 2.7	
							**-ADAS-cog**:(A) 17.7 ± 1.2 (B) 19.4 ± 1.4	
	116 (58:58)	(B) 65.33 ± 10.24	(B) 30:28		Galantamine, 8 mg, bid		**-Blessed-Roth**:(A) 19.4 ± 3.6 (B) 20.4 ± 2.6	
							**-CER (ng/L**):(A) 0.23 ± 0.05 (B) 0.20 ± 0.08	
							**-BDNF (pg/mL**):(A) 0.23 ± 0.05 (B) 0.21 ± 0.02	
							**-miR.132**:(A) 0.74 ± 0.18 (B) 0.62 ± 0.25	
Zhou^[26]^	64 (32:32)	(A) 70.63 ± 2.37	(A) 16:16	AD (CCMD)**-MMSE**:(A) 6.85 ± 1.36(B) 6.71 ± 1.25	(B) + Magnesium valproate sustained-release tablets 0.25 mg qd, after 1 week of treatment, the dose was increased to 5–10 mg qd	3 m	**-MMSE**:(A) 27.52 ± 2.41(B) 19.37 ± 3.88	(A):2 (6.25)(B):4 (12.5)
	64 (32:32)	(B) 71.06 ± 2.43	(B)17:15		Donepezil 2.5–5 mg qd, after 1 week of treatment, the dose was increased to 5–10 mg qd			
Zhang^[27]^	30 (15:15)	(A) 73.1 ± 1.6	(A) 9:6	AD (CCMD-3)**-MMSE**:A) 16.5 ± 6.9(B) 16.6 ± 7.1	(B) + Magnesium valproate sustained-release tablets 0.25 mg qd, after 1 week of treatment, the dose was increased to 0.5 mg qd	1 m/3 m	**-MMSE**:(A) 18.0 ± 5.1/2.3 ± 5.1(B) 17.3 ± 4.7/19.7 ± 3.9	(A):2 (13.3)(B):3 (20)
	30 (15:15)	(B) 72.1 ± 1.8	(B) 8:7		Donepezil 2.5–5 mg qd, after 1 week of treatment, the dose was increased to 5–10 mg qd		**-BEHAVE-AD:**(A) 12.5 ± 3.8/6.2 ± 2.8(B) 13.8 ± 4.0/10.1 ± 3.4	
Liu et al^[28]^	60 (30:30)	(A) 71.54 ± 3.26	(A) 17:13	AD (CCMD)**-HAMD** ≤ 17**-MMSE**:(A) 23.18 ± 2.36(B) 22.78 ± 2.35	(B) + Magnesium valproate sustained-release tablets 250 mg bid	3 m	**-MMSE**:(A) 9.62 ± 1.32 (B) 16.83 ± 1.03	NA
							**-ADL**:(A) 11.32 ± 1.31 (B) 14.26 ± 1.54	
	60 (30:30)	(B) 73.26 ± 3.15	(B) 16:14		Donepezil 5 mg qd, after 1 week of treatment, the dose was increased to 10 mg qd		**-ADAS**:(A) 8.69 ± 1.21 (B) 15.23 ± 1.24	
							**-PSQI**:(A) 9.85 ± 1.26 (B) 14.58 ± 1.39	
Cong et al^[29]^	120 (60:60)	(A) 72 ± 5	(A) 28:32	AD (DSM)**-MMSE**:(A) 18.4 ± 2.8(B) 18.5 ± 3.1	(B) + Magnesium valproate sustained-release tablets 0.25 mg qd	8 w	**-MMSE**:(A) 24.0 ± 4.73 (B) 20.4 ± 4.3	(A):6 (10.1)(B):10 (16.7)
							**-ADL**:(A) 19 ± 5 (B) 26 ± 6	
							**-NPI**:(A) 25 ± 9 (B) 19 ± 9	
	120 (60:60)	(B) 73 ± 5	(B) 33:27		Donepezil, 5 mg, qd		**-IL-6 (pg/L**):(A) 154 ± 14 (B) 174 ± 17	
							**-TNF-α (pg/L**):(A) 84 ± 19 (B) 138 ± 38	
							**-CRP (mg/L**):(A) 3.3 ± 0.8 (B) 6.6 ± 1.4	
Fang^[30]^	76 (38:38)	(A) 72.8 ± 7.1	(A) 21:17	AD (CCMD)**-MMSE**:(A) 6.53 ± 1.38(B) 6.56 ± 1.43	(B) + Magnesium valproate sustained-release tablets 0.25 mg qd, after 1 week of treatment, the dose was increased to 5–10 mg qd	3 m	**-MMSE**:(A) 26.35 ± 6.36(B) 19.87 ± 6.28	(A):3 (7.89)(B):5 (13.15)
	76 (38:38)	(B) 72.1 ± 7.5	(B) 20:18		Donepezil 2.5–5 mg qd, after 1 week of treatment, the dose was increased to 5–10 mg qd		**-ADL**:(A) 25.46 ± 4.31(B) 34.72 ± 4.79	
Hao and Yang^[31]^	76 (36:36)	(A) 65.3 ± 8.4	(A) 19:17	AD (ICD-10)**-BEHAVE-AD** ≥ 8	(B) + Magnesium valproate sustained-release tablets,The initial dose was 250 mg/d and the therapeutic dose was 250–1000 mg/d	2 w/4 w/8 w	**-BEHAVE-AD:**(A) 14.5 ± 4.0/10.2 ± 4.0/6.8 ± 2.8 (B) 16.8 ± 4.4/12.6 ± 3.8/7.2 ± 2.1	(A):13 (37.1)(B):7 (20.6)
	69 (34:35)	(B) 66.4±6.7	(B) 21:15	VD (ICD-10)	Quetiapine 50 mg/d, after 4 days of treatment, the dose was increased to 200–400 mg/d			
Li^[32]^	56 (28:28)	(A) 72.15 ± 6.3	(A) 10:18	AD**-MMSE** < 24**-BRMS** > 10	(B) + Magnesium valproate sustained-release tablets 0.2–1.0 g/d	1 w/2 w/4 w	**-BRMS**:(A) 15.24 ± 1.88/10.74 ± 1.67/ 7.23 ± 1.91 (B) 18.66 ± 2.56/14.43 ± 2.11/8.01 ± 1.64	Unclear
	56 (28:28)	(B) 73.42 ± 5.5	(B) 9:19		Olanzapine, 2.5 mg/d, gradually increase the dose to 15 mg qd			
Sun et al^[33]^	70 (35:35)	71.3 ± 3.5	43:27	AD**-HAMD** ≤ 17**-HIS** < 4**-MMSE**:(A) 2.2 ± 1.5(B) 4.5 ± 2.3	(B) + Magnesium valproate sustained-release tablets 0.25 mg qd, after 1 week of treatment, the dose was increased to 0.5 mg qd	2 w/16 w	**-MMSE**:(A) 18.3 ± ± 6.8/26.4 ± 6.2(B) 15.7 ± ± 7.3/21.5 ± 6.4	(A):4 (12.5)(B):8 (28.1)
	64 (32:32)				Donepezil 2.5–5 mg qd, after 1 week of treatment, the dose was increased to 5–10 mg qd			
Zhou^[34]^	102 (51:51)	(A) 60.12 ± 4.98	(A) 21:30	AD (The 5th edition of the Psychiatry Book):**-BRMS >** 10**-MMSE** < 24:(A) 13.08 ± 2.41(B) 13.21 ± 2.05	(B) + Magnesium valproate sustained-release tablets 0.2 g/d qd	6 w	**-MMSE**:(A) 21.24 ± 2.87 (B) 18.03 ± 3.10	(A):8 (15.69)(B):10 (19.61)
	102 (51:51)	(B) 60.23 ± 4.44	(B) 18:33		Olanzapine, 2.5 mg/d, gradually increase the dose to 15 mg qd		**-ADAS-cog**:(A) 23.5 ± 2.14(B) 28.11 ± 2.29	
Fan^[35]^	64 (32:32)	70.2 ± 2.5	46:18	AD (CCMD)**-HAMD** ≤ 17**-HIS** < 4**-MMSE**:(A) 31.18 ± 9.78(B) 36.00 ± 6.39	(B) + Magnesium valproate sustained-release tablets 0.25 mg qd, after 1 week of treatment, the dose was increased to 0.5 mg qd	2 w/16 w	**-MMSE**:(A) 27.40 ± 9.27/4.42 ± 5.66(B) 29.62 ± 7.73/2.15 ± 4.52	(A):4 (12.5)(B):9 (28.1)
	64 (32:32)				Donepezil 2.5–5 mg qd, after 1 week of treatment, the dose was increased to 5–10 mg qd			
ZhangandWang^[36]^	70 (35:35)	(A) 61–82	(A) 19:16	AD (CCMD-3)**-BRMS >** 10**-MMSE** < 24	(B) + Magnesium valproate sustained-release tablets 0.25–1.0 g/d	1 w/2 w/4 w/6 w	**-BRMS**:(A) 15.67 ± ± 1.79/11.40 ± 1.89/9.33 ± 1.90/6.23 ± 2.08 (B) 18.71 ± 2.42/15.13 ± 2.23/12.40 ± 1.64/8.42 ± 1.6	Unclear
	70 (35:35)	(B) 64–82	(B) 15:20		Olanzapine, 2.5 mg/d, gradually increase the dose to 15 mg qd			
Yao^[37]^	65 (30:35)	Unclear	Unclear	AD (CCMD-3)**-MMSE** ≤ 24**-BEHAVE-AD** ≥ 8	Aripiprazole, (6 ± 2.2) mg/d + VPM, (0.5 ± 0.3) g/d	1 w/2 w/4 w/6 w/8 w	**-CMAI**: (A) 51.9 ± 11.5/49.3 ± 12.6/47.5 ± 11.8/40.1 ± 12.5/37.5 ± 13.6 (B) 51.4 ± 11.8/50.2 ± 12.8/48.4 ± 12.8/42.2 ± 13.6/38.6 ± 14.6	NA
	65 (30:35)				Aripiprazole (8 ± 2.8) mg/d		**-BEHAVE-AD:**(A) 15.6 ± 7.4/8.8 ± 7.8/8.6 ± 6.4/6.8 ± 5.3/6.2 ± 4.2 (B) 15.6 ± 7.4/15.8 ± 7.8/10.6 ± 6.4/6.8 ± 4.2/6.2 ± 4.2	
Xie^[38]^	51 (26:25)	(A) 66.8 ± 12.3	(A) 12:14	AD (CCMD-3)-HIS < 4**-BEHAVE-AD** ≥ 8**-MMSE** < 24:(A) 9.2 ± 3.4(B) 8.9 ± 3.9	Olanzapine, (6.5 ± 5.4) mg/d + Magnesium valproate sustained-release tablets, 0.25–1.0 g/d	2 w/4 w/6 w	**-MMSE**:(A) 9.5 ± 1.6/10.0 ± 1.1/10.8 ± 2.4 (B) 9.3 ± 2.1/9.9 ± 2.6/10.5 ± 3.1	(A):11 (42.3)(B):17 (68)
	51 (26:25)	(B6) 7.3 ± 8.8	(B) 11:14		Olanzapine, (9.4 ± 6.8) mg/d			

### 2.4. Quality assessment

Two reviewers, respectively, used the bias risk assessment guideline (recommended by the Cochrane handbook, http://community.cochrane.org/handbook) for quality assessment in the included 22 RCTs. The domains including the following 7 aspects, include random sequence generation, allocation concealment, blinding of the participants, blinding of outcome assessments, incomplete outcome data, and selective outcome reporting. The reviewers were blinded to each other’s results. The results were then analyzed by level of agreement between the 2 reviewers. There were any disagreements between the 2 examiners were deal with a third examiner and the study will be reevaluated then.

### 2.5. Statistical analysis

Stata 16.0 software was performed for our statistical analyses. Standardized mean difference with 95% CI as an effect size was measured for continuous data. As far as dichotomous data, the risk ratio (RR) with 95% CI was calculated. Cochran Q statistic and I2 metric statistics were used to assess the level of heterogeneity. Among them, the Q test is used to assess the presence of heterogeneity, and the I2 index is used to quantify the extent of heterogeneity. According to the values of I2 50% and > 50%, heterogeneity was classified as being with or without significant heterogeneity. In this study, due to the significant heterogeneity (I2 > 50%, *P* < .05), we performed a random-effects model with analyze of different scale assessments about cognitive, psychiatric symptoms, and relevant serum indicators. A fixed-effects model was used to calculate the rate of adverse reactions while I2 < 50%. All tests in our statistical analyses were 2-sided and statistical significance was indicated when *P* < .05. We considerate to applying sensitivity analysis to test the stability of the results and funnel plots to examine potential publication bias where it was necessary.

## 3. Results

### 3.1. Included studies

**Figure 1.**
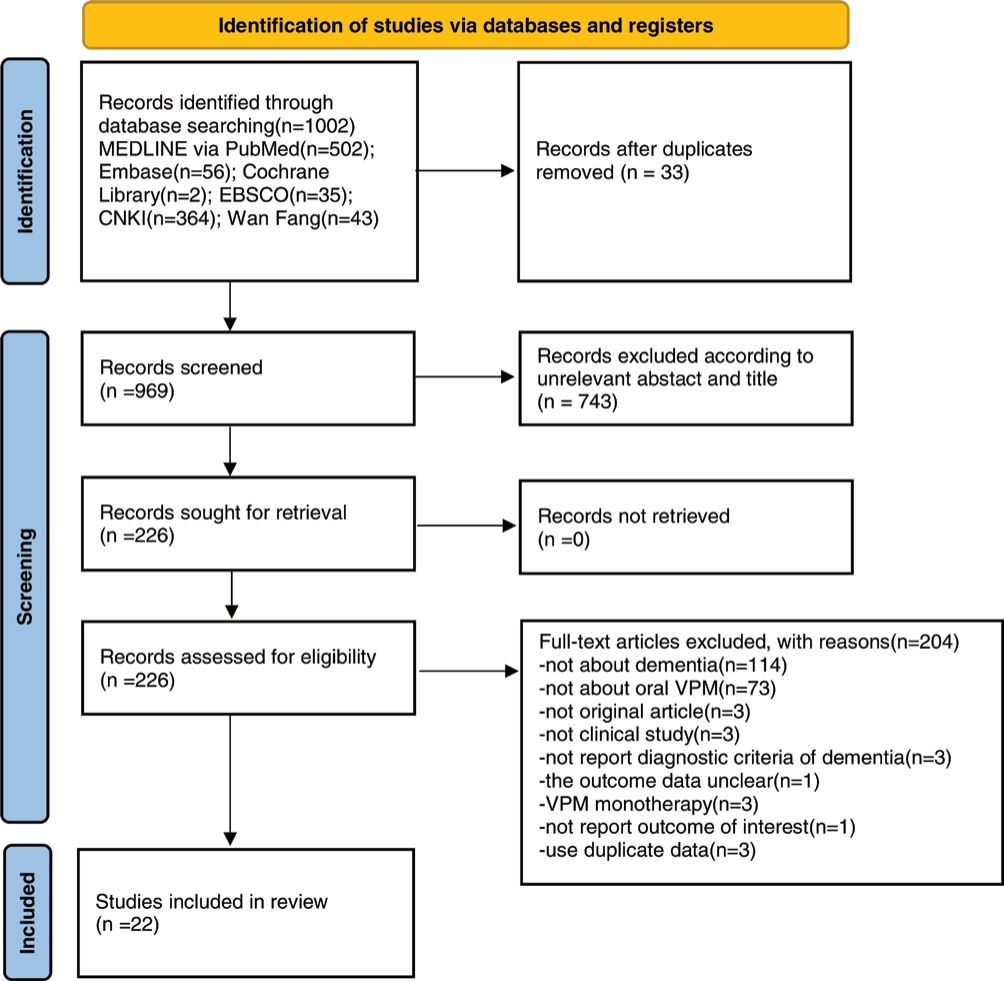
A PRISMA flow diagram of the literature screening and selection processes. EBSCO, EltonB. Stephens Company. CNKI = China National Knowledge Infrastructure, VPM = valproic acid magnesium.

A total of 1002 records were retrieved from electronic database searches. As shown in **Figure 1**, 969 articles remained after the relevant duplicates were removed. Via screening of titles and abstracts, the full papers of 226 articles were obtained and evaluated them for eligibility. Finally, based on the inclusion and exclusion criteria, 22 RCT studies (a total of 1899 participants) that presented basic characteristics and outcome about VPM-assistant therapy on dementia were included (Table 1). It is worth noting that when the outcome scores of different treatment time were included in 1 RCT, they were calculated as different trails results.

### 3.2. Quality assessment of included studies

**Figure 2.**
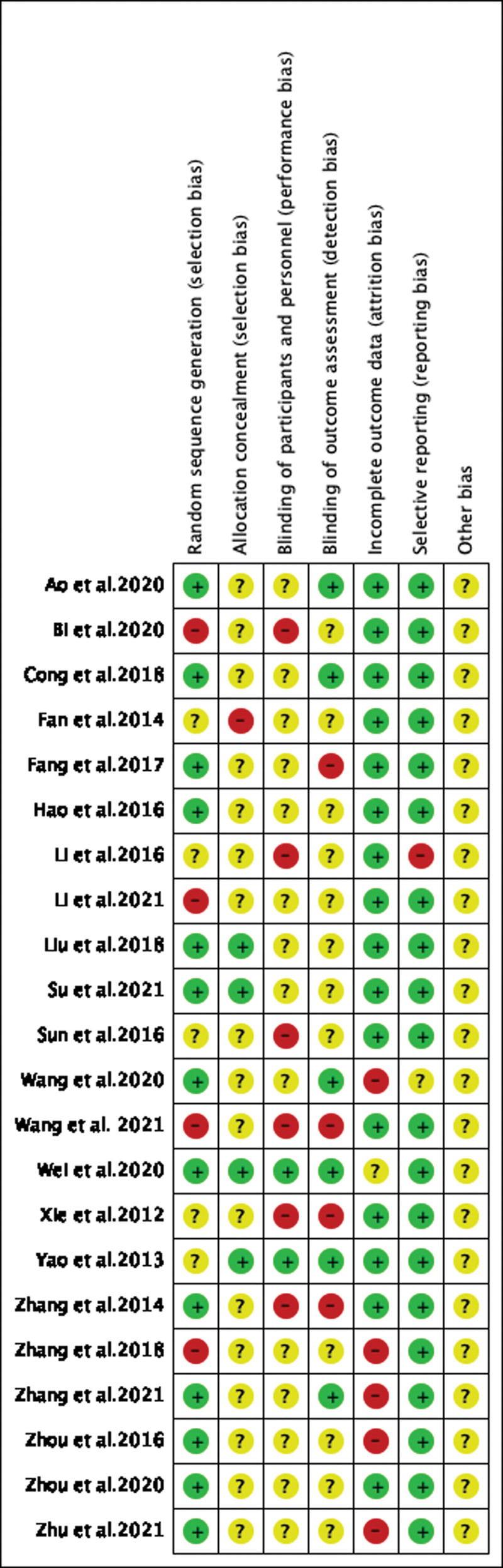
Risk of bias of all included studies. Low, unclear, and high risk, respectively, are represented with the following symbols: “+”, “?”, “–.”

The final results of quality assessments of 22 studies are summarized in **Figure 2**.

### 3.3. Efficacy in cognitive effects

**Figure 3.**
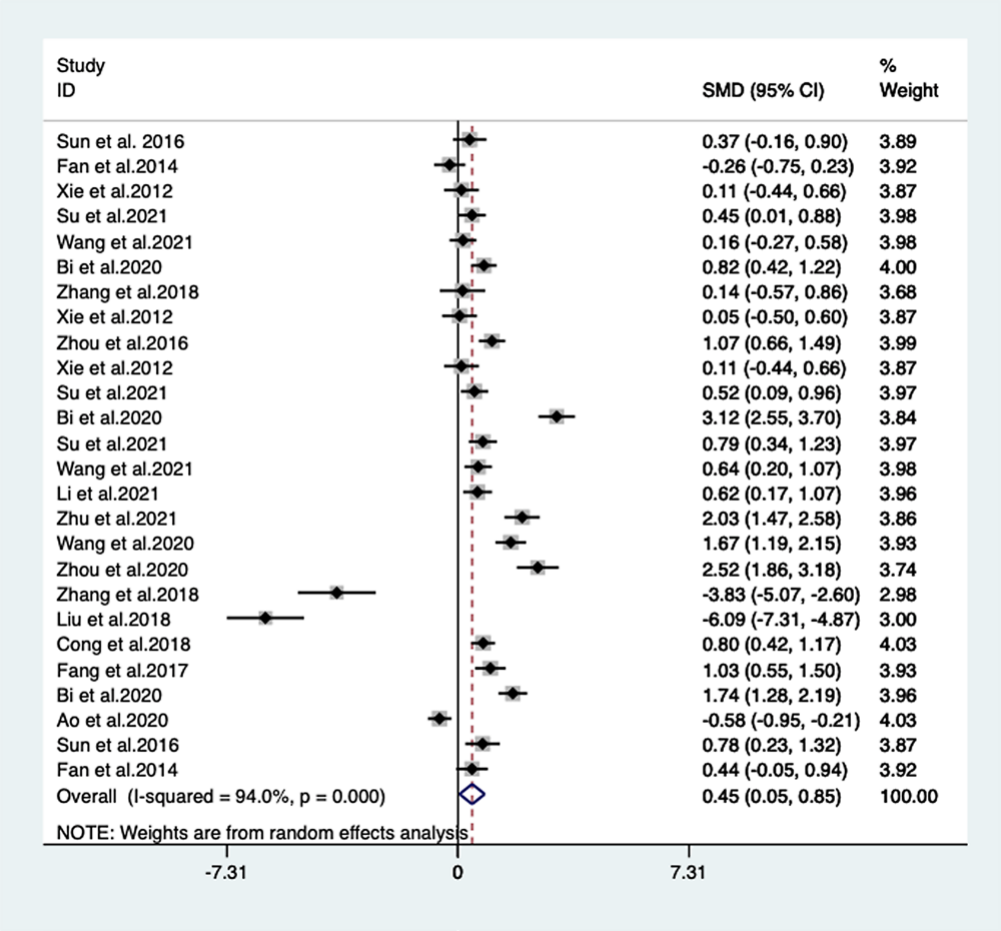
MMSE score between the VPM adjuvant treatment group with control group (SMD = 0.447, 95% CI: 0.049 to 0.846, *P* = .028). CI = confidence interval, MMSE = Mini-mental State Examination, SMD = standardized mean difference, VPM = magnesium valproate.

When VPM was additionally used as an antidementia drug, cognitive effects were measured by Mini-mental State Examination (MMSE) in 16 articles^[15,18–20,23–30,33–35,38]^ (included 26 trails) with 1971 patients comparing with monotherapy group, by meta-analysis, the MMSE score (SMD = 0.447, 95% CI: 0.049 to 0.846, *P* = .028) was significantly improved, but with significant heterogeneity (level of heterogeneity χ^2^ = 418.79, df = 25, *P* < .001, I^2^ = 94%) (**Fig. 3**).

**Figure 4.**
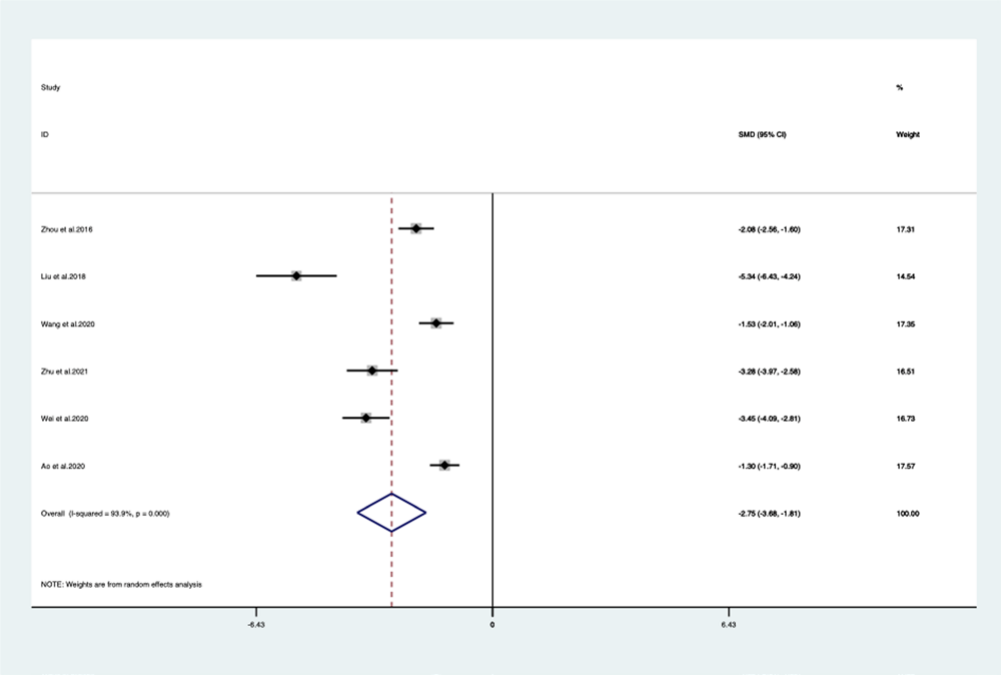
ADAS-cog score between the VPM adjuvant treatment group with control group (SMD –2.749, 95% CI: –3.684 to –1.813, *P* < .05). ADAS-cog = Alzheimer disease assessment scale–cognitive subscale, CI = confidence interval, SMD = standardized mean difference, VPM = magnesium valproate.

In addition to, ADAS-cog applied in 6 RCTs^[15,22,23,25,28,34]^ with 538 patients in VPM group was significantly lower than control group (SMD –2.749, 95% CI: –3.684 to –1.813, *P* < .05) (**Fig. 4**). However, the MOCA score of 2 RCTs^[20,25]^ was not show any significant difference between VPM-assisted therapy group with antidementia drugs alone (SMD 0.09, 95% CI: –1.879 to –2.059, *P* = .929).

### 3.4. Efficacy in psychiatric effects

**Figure 5.**
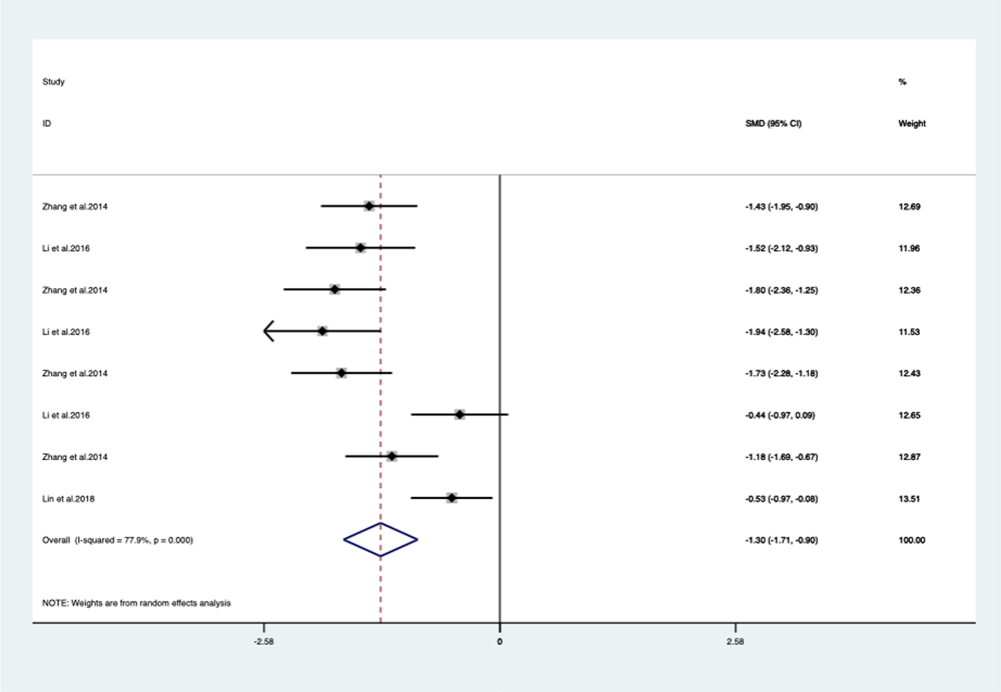
BRMS score between the VPM adjuvant treatment group with control group (SMD –1.303, 95% CI: –1.709 to –0.898, *P* < .05). BRMS = Bech-Rafaelsen Mania Rating Scale, CI = confidence interval, SMD = standardized mean difference, VPM = magnesium valproate.

**Figure 6.**
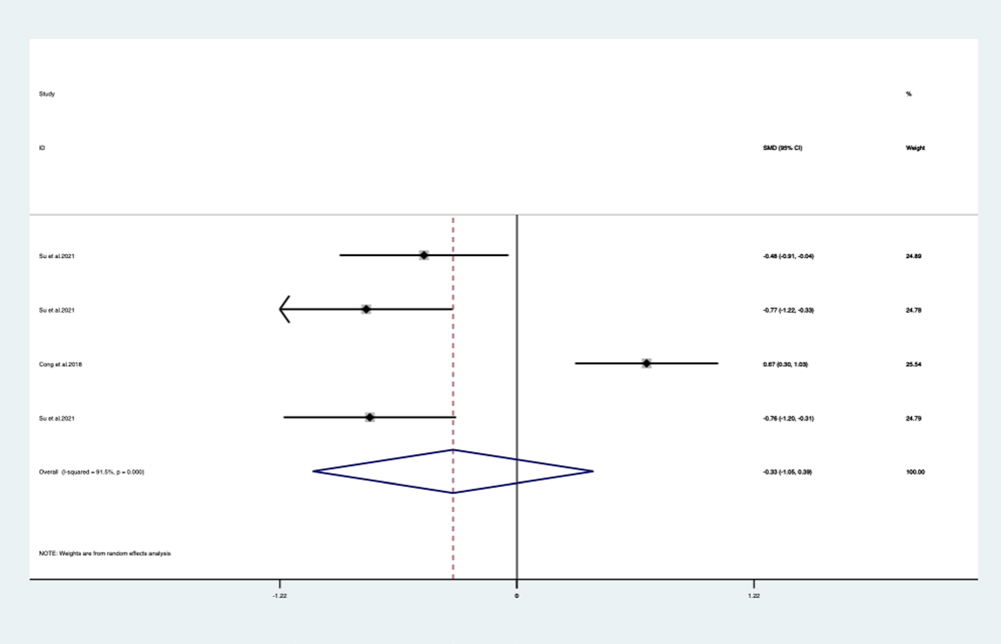
NPI score between the VPM adjuvant treatment group with control group (SMD –0.328, 95% CI: –1.049 to 0.393, *P* = .373). CI = confidence interval, NPI = neuropsychiatric inventory, SMD = standardized mean difference, VPM = magnesium valproate.

**Figure 7.**
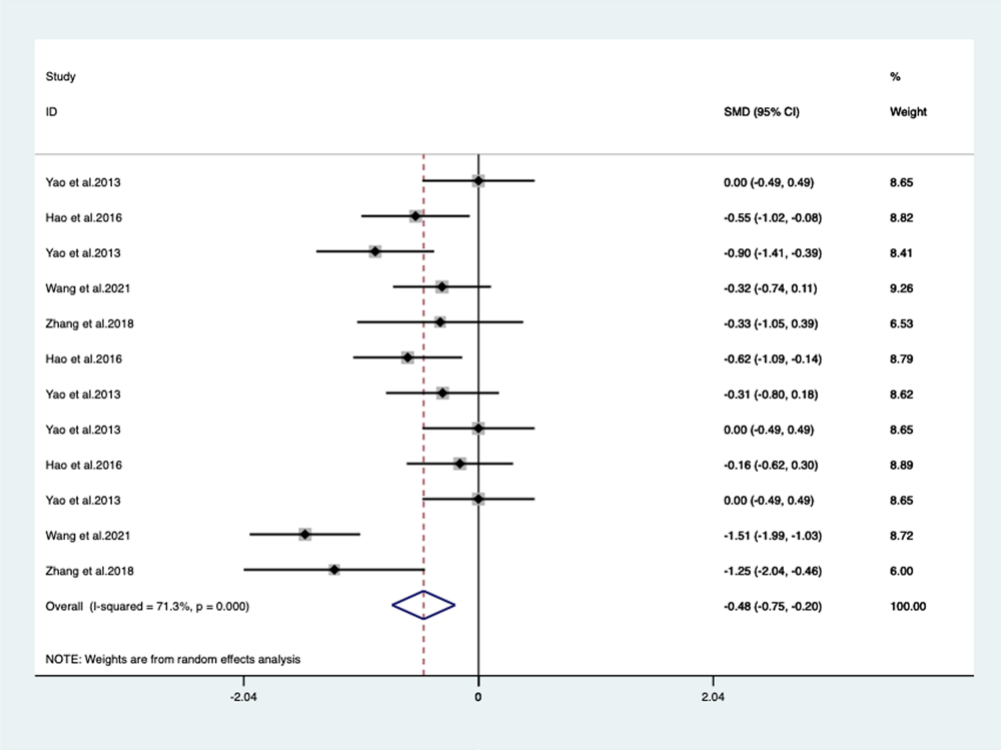
BEHAVE-AD score between the VPM adjuvant treatment group with control Group (SMD –0.477, 95% CI: –751 to 0.203, *P* = .001). BEHAVE-AD = behavioral pathology in Alzheimer disease rating scale, CI = confidence interval, SMD = standardized mean difference, VPM = magnesium valproate.

When VPM was additionally used for psychotropic drugs, the severity of behavioral and psychological symptoms of dementia measured by BRMS in 3 articles^[28,32,36]^ (included 8 trails) with 532 patients (SMD –1.303, 95% CI: –1.709 to –0.898, *P* < .05) (**Fig. 5**) significantly improved compared with psychotropic drugs alone. Agitation was evaluated by additional specific scales included Neuropsychiatric Inventory (NPI) and Cohen-Mansfield Agitation Inventory. By meta-analysis, the NPI change of score was show no significant difference between the 2 studies^[18,29]^ (SMD –0.328, 95% CI: –1.049 to 0.393, *P* = .373) (**Fig. 6**) but 1 study Yao et al^[37]^ reported that the Cohen-Mansfield Agitation Inventory score improved significantly (*P* < .05). When VPM was additionally used, significant difference in our meta-analysis was also found between VPM group and control group of BEHAVE-AD score in 4 articles^[19,27,31,37]^ (included 12 trails) with 773 patients (SMD –0.477, 95% CI: –751 to 0.203, *P* = .001) (**Fig. 7**).

### 3.5. Efficacy in Inflammatory factor levels

**Figure 8.**
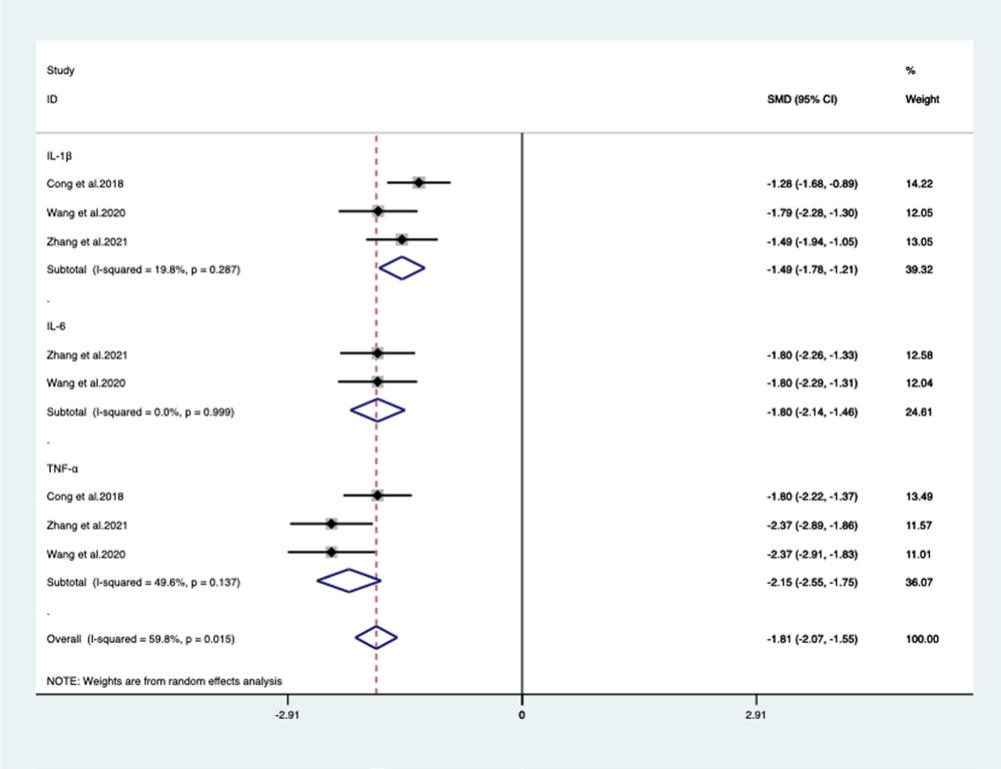
Inflammatory factor levels between the VPM adjuvant treatment group with control group. VPM = magnesium valproate.

Inflammatory factor levels including IL-1β, IL-6, TNF-α of PwD were also discussed. IL-1β was applied in 3 RCTs^[21,23,29]^ with 310 patients (SMD –1.494, 95% CI: –1.777 to –1.211, *P* < .05), IL-6 was applied in 2 RCTs^[21,23]^ with 190 patients (SMD –1.797, 95% CI: –2.135 to –1.459, *P* < .05) and TNF-α was applied in 3 RCTs^[21,23,29]^ with 310 patients (SMD –2.153, 95% CI: –2.553 to –1.753, *P* < .05), respectively. The results all showed significant difference found in VPM-assisted therapy group versus control group (**Fig. 8**).

### 3.6. Efficacy in other outcomes

**Figure 9.**
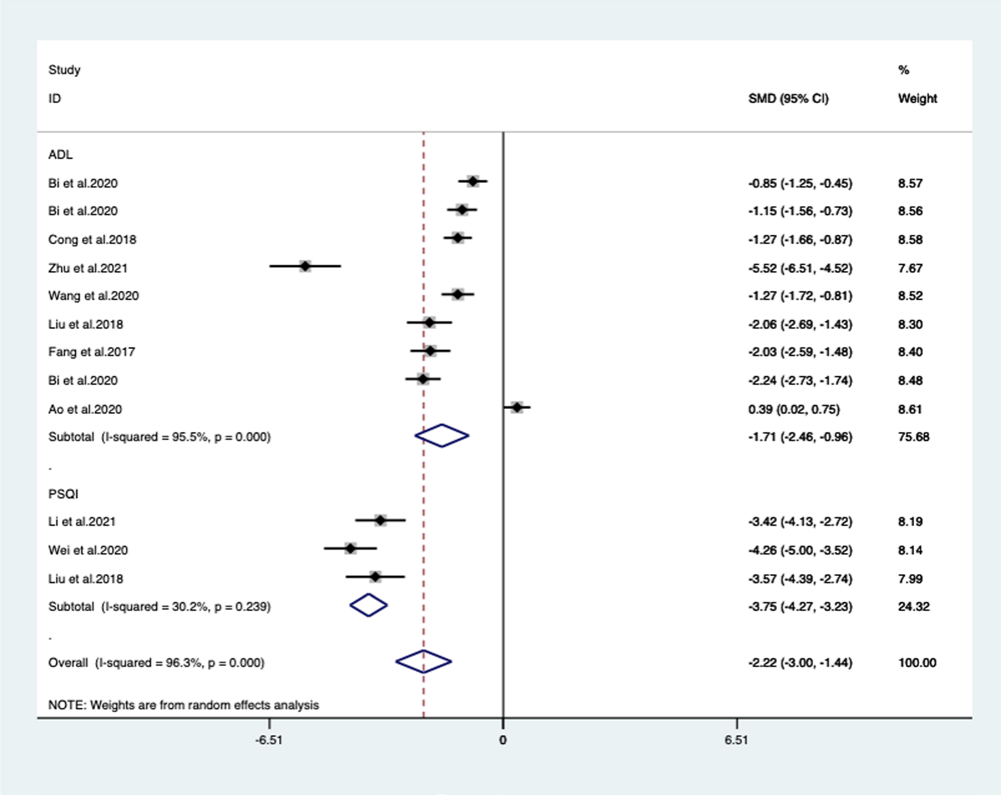
ADL and PSQI scores between the VPM adjuvant treatment group with control group. ADL = activities of daily living, PSQI = Pittsburgh sleep quality index, VPM = magnesium valproate.

When VPM was additionally used, other outcomes including ADL in 7 articles^[15,23–25,28–30]^ (included 9 trails) with 850 patients (SMD –1.711, 95% CI: –2.459 to –0.962, *P* < .05) and PSQI in 3 articles^[20,22,28]^ with 232 patients (SMD –3.751, 95% CI: –4.270 to –3.232, *P* < .05) both significantly improved, compared with psychotropic drugs or antidementia drugs alone (**Fig. 9**).

### 3.7. Safety

**Figure 10.**
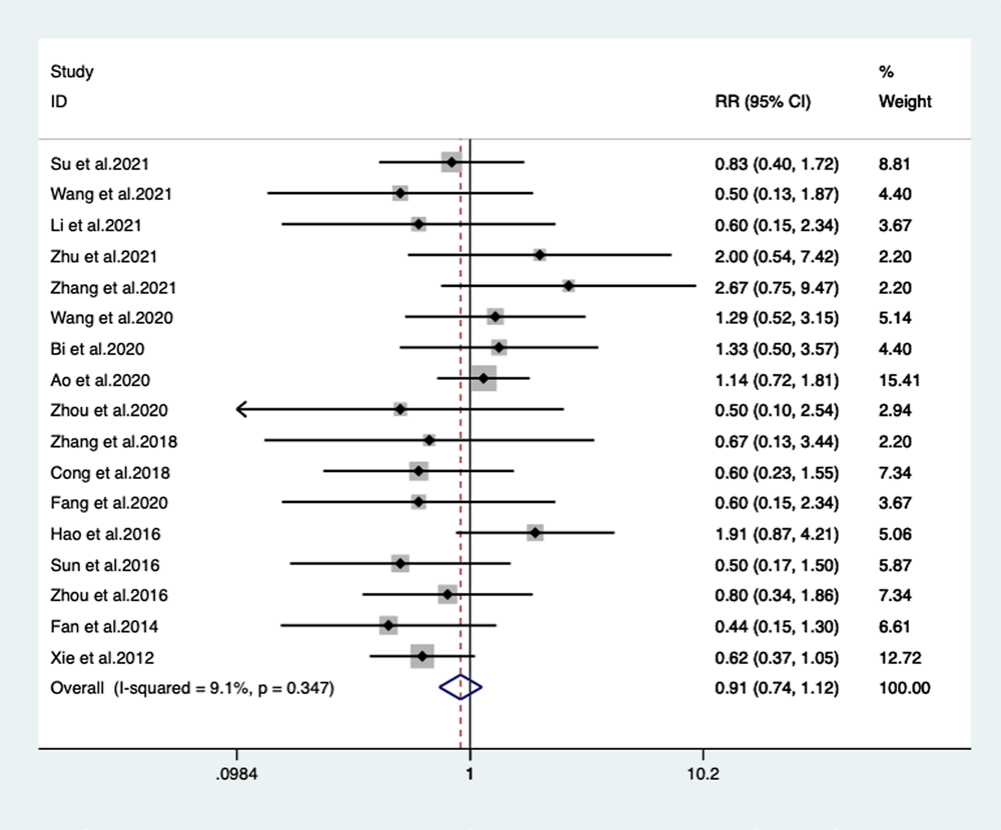
Adverse events between the VPM adjuvant treatment group with control Group (RR 0.910, 95% CI: 0.736–1.125, *P* = .383). CI = confidence interval, RR = risk ratio, VPM = magnesium valproate.

Seventeen RCTs^[15,18–21,23–27,29–31,33–35,38]^ with 1374 patients reported the number of patients with adverse events. By our meta-analysis, when compared with monotherapy group, there were no significant differences found in VPM group (RR 0.910, 95% CI: 0.736 to 1.125, *P* = .383) (**Fig. 10**).

### 3.8. Subgroup

Considering the design of the included studies, the severity of dementia was not included in all studies, so we cannot perform a subgroup analysis of dementia severity. In addition, the dose of VPM used in these studies was almost consistent, and there was also no relevant analysis of drug dose. Based on the follow-up time, subgroup analysis was performed, MMSE score at duration times of < 12 weeks (SMD = 0.555, 95% CI: 0.110 to 0.999, *P* = .014) was significantly improved while compared with control group, MMSE score was not significantly different for durations ≥ 12 weeks (SMD = 0.309, 95% CI: –0.345 to 0.963, *P* = .354). As well as other outcomes, subgroup analysis were also performed, based on duration of treatment (**Table 2**).

**Table 2:** Subgroup analysis based on duration time

Outcomes	Subgroup	No.articles/No.RCTs	No. participants	Effect estimate SMD/RR [95% CI]	I^2^ value (%)	*P* (Test of SMD/RR)
**Cognitive assessment**						
MMSE	Total	16/26	1971	SMD 0.447 [0.049, 0.846]	94	0.028
Duration time	<12 w	8/12	867	SMD 0.555 [0.110, 0.999]	89.9	0.014
	≥12 w	8/14	1104	SMD 0.309 [–0.345, 0.963]	95.8	0.354
MOCA	Total	2/2	194	SMD 0.09 [–1.879, –2.059]	97.6	0.929
ADAS-cog	Total	6/6	538	SMD –2.749 [–3.684, –1.813]	93.9	<0.05
duration time	<12 w	1/1	102	SMD –2.080 [–2.564, –1.597]	NA	0.014
	≥12 w	5/5	436	SMD –2.911 [–4.107, –1.716]	95.1	0.354
**Psychiatric assessment**						
BRMS	Total	3/8	532	SMD –1.422 [–1.802, –1.041]	77.9	<0.05
Duration time	<12 w	2/7	448	SMD –0.526 [–0.972, –0.080]	69.5	<0.05
	≥12 w	1/1	84	SMD –2.911 [–4.107, –1.716]	NA	0.021
BEHAVE-AD	Total	4/12	773	SMD –0.477 [–0.751, 0.203]	71.3	0.001
duration time	<12 w	4/10	657	SMD –0.315 [–0.500, –0.130]	29	0.001
	≥12 w	2/2	116	SMD –1.438 [–1.848, –1.028]	NA	<0.05
NPI	Total	2/4	372	SMD –0.328 [–1.049, 0.393]	91.5	0.373
duration time	<12 w	2/3	288	SMD –0.188 [–1.090, –0.714]	93	0.683
	≥12 w	1/1	84	SMD –0.756 [–1.199, –0.313]	NA	0.001
**Inflammatory factor levels**						
IL-1β	Total	3/3	310	SMD –1.494 [–1.777, –1.211]	19.8	< 0.05
duration time	<12 w	1/1	120	SMD –1.284 [–1.678, –0.891]	NA	< 0.05
	≥12 w	2/2	190	SMD –1.628 [–1.958, –1.299]	0	< 0.05
IL-6	Total	2/2	190	SMD –1.797 [–2.135, –1.459]	NA	< 0.05
TNF-α	Total	3/3	310	SMD –2.153 [–2.553, –1.753]	49.6	< 0.05
duration time	<12 w	1/1	120	SMD –1.798 [–2.223, –1.372]	NA	< 0.05
	≥12 w	2/2	190	SMD –2.372 [–2.745, –2.000]	0	< 0.05
**Other outcomes**						
ADL	Total	7/9	850	SMD –1.711 [–2.459, –0.962]	95.5	< 0.05
duration time	<12 w	2/3	328	SMD –1.088 [–1.335, –0.841]	11.2	< 0.05
	≥12 w	5/6	522	SMD –0.386 [0.018, 0.753]	97.2	0.001
PSQI	Total	3/3	232	SMD –3.751 [–4.270, –3.232]	30.2	< 0.05
**Safety**						
Adverse event	Total	17/17	1374	RR 0.910 [0.736, 1.125]	9.1	0.383

## 4. Discussion

Our interest in VPM as an adjuvant therapy in PwD stems from rational mechanisms of action and the lack of therapies that balance efficacy and safety. This meta-analysis identified 22 RCTs aiming to test the effect of VPM adjuvant therapy in the treatment of dementia. Conducted than other studies in recent years, it first provides the effect about VPM-assistant therapy with PwD, and the results not only limited on psychiatric symptoms, but also offer more evidence to other reviews, for example, inflammatory factor levels, which is relatively objective in compare with neuropsychological tests.

Previously, there were several studies suggesting that valproic acid was ineffective^[13,14]^ even negative results in treating agitation among demented patients.^[15]^ According to our findings, we found that VPM as an adjuvant therapy had a general positive effect on the cognitive function and psychiatric symptoms of PwD, including the MMSE, ADAS-cog, BEHAVE-AD, and BRSM score, especially when VPM was additionally used within 12 weeks, MMSE score improved obviously. VPM, indeed, which is a most used antiepileptic drugs (AEDs) and safe in patients with epilepsy (PwE) under the long duration treatment. The pharmacological effects of VPM are mainly competitive inhibition of γ-aminobutyric acid transferase, regulation of γ -aminobutyric acid metabolism in the brain, to improve the content of γ-aminobutyric acid in the central nervous system. γ-aminobutyric acid can promote brain cell metabolism, improve nerve function, and memory disorders.^[39]^ Magnesium supplementation in the diet has been shown to improve memory in AD. The “PATH through Life” Project conducted by Cherbuin et al^[40]^ found that higher magnesium intake was related to a reduced risk of developing mild cognitive impairment and mild cognitive disorders. In addition, the suggested mechanisms by which valproic acid may have an impact on agitation include enhancement of the intracerebral neurotransmission agent, GABA, antimanic action, and effect on mood stabilizing.^[41]^ As far as the individual study, there was no evidence of a beneficial effect of valproate on agitation or closely related behavioral outcomes^[13,14]^ and conflicting results were seen in our study.

The results of neuroprotective effects not only include improvements in cognitive function or psychiatric symptoms, but also improvements in quality-of-life, disease modifying, even survival, and other functional abilities. Thus, it can be observed that the ADL and PSQI were significantly improved in the VPM group in this study, which is different to the previous systematic review. Actually, cognitive improvement and disease progression are still the most important indicators for PwD in terms of survival time and quality-of-life. Unfortunately, in our study, we still did not refer to the outcome of other changes, such as survival, which is the same as in other studies. The short follow-up time may be the main factor.

Besides neuropsychological tests, we also conducted an analysis of serum inflammatory factors. IL-1β, IL-6, and TNF-α were all decreased significantly when VPM used additional. In fact, compared with neuropsychological tests, biomarkers are relatively objective in illustrating the neurodegenerative process. According to laboratory studies, microglia and astrocytes in AD patients overexpress proinflammatory cytokines, while IL-1β, IL-6, and TNF-α accelerate the progression of AD and inhibit the transmission of cholinergic neurons, ultimately leading to central nervous system damage.^[42]^ VPM can effectively reduce neuronal injury in patients, and play a protective role in brain by affecting protein kinase pathways, transcription factors, and promoting nerve regeneration, thereby reducing the level of inflammatory factors.^[43]^ Therefore, we consider that this may be one of the potential mechanisms of VPM-assisted therapy for dementia patients.

However, our study also had several limitations. First, most of these experiments were carried out in China, which may be because magnesium valproate is a common drug in China, while sodium valproate is widely used in Western countries, which may lead to regional bias. Second, there was lack of classification of dementia severity in our inclusion study, so we could not make a subgroup analysis of dementia severity. Third, the observation time of these studies is short, even though the longest period is only 16 W, which lacks analysis of the prognosis and survival rate of later stage of disease. Besides, in the included trials, some of indicators showed a high percentage of heterogeneity, therefore, we need to be cautious when drawing general conclusions.

With consideration in mind about the question “What is the effect of VPM on PwD?” we can discuss it from the following perspectives in future research:

- Future clinical studies are needed to supplement the literature on whether magnesium or VPM should be a complementary treatment option for PwD.
- Clinical trials of VPM long-term adjunctive therapy were designed to determine whether disease-modifying therapy in PwD was justified and needed.
- It is reasonable that studies designed for VPM-assisted therapy need to be carefully evaluated, including sample size, participants, severity of dementia, duration of treatment, and adverse effects

## 5. Conclusion

VPM as assistant therapy is generally well tolerated in PwD. In our study, subjective scales and objective serum indicators showed that magnesium valproate adjuvant therapy had a positive impact on cognitive function, psychiatric symptoms, and disease prognosis of dementia patients, without increasing adverse events. However, much more studies are still needed regarding magnesium or magnesium valproate or its corresponding type of sustained-release tablet effects on PwD, including clinical assessment the use of VPM as a complementary treatment.

## Author contributions

ChenQi Zhang and HongBin Sun designed the experiments. ChenQi Zhang and LingQi Sun conducted literature screening and data extraction. HongBin Sun supervised the data collection. ChenQi Zhang wrote the initial draft. All authors contributed to the article and approved the submitted version.

## Supplementary Material


