# Supplementary material for: Effects of magnesium valproate adjuvant therapy on patients with dementia: A systematic review and meta-analysis
Source: Medicine (Baltimore). 2022 Aug 5;101(31):e29642. doi: 10.1097/MD.0000000000029642 (PMC9351853; doi:10.1097/MD.0000000000029642)
Supplement: Supplementary file 1 [file medi-101-e29642-s001.pdf]

## Supplement 1. Search terms used in each database

### MEDLINE via PubMed

| | Searches | Results |
|---|---|---|
| #1 | ((((Valproate Magnesium[MeSH Terms]) OR (Valproic acid magnesium)) OR (Magnesium dipropylacetate)) OR (Magnesium valproate sustained release agent)) OR (Magnesium 2-propylvalerate) | 16,581 |
| #2 | ((((((((((Dementia[MeSH Terms]) OR (dement*[Title/Abstract])) OR (Alzheimer*[Title/Abstract])) OR (Huntington*[Title/Abstract])) OR (Parkinson*[Title/Abstract])) OR ("Lewy body"[Title/Abstract])) OR ("Pick disease"[Title/Abstract])) OR ("cognitive impairment"[Title/Abstract])) OR ("vascular dementia"[Title/Abstract])) OR ("neurodegenerative disease"[Title/Abstract])) OR ("neurocognitive disorder"[Title/Abstract]) | 432,291 |
| #3 | #1 AND #2 | **502** |

### EMBASE

| | Searches | Results |
|---|---|---|
| #1 | 'valproate magnesium'/exp OR (valproic AND acid AND magnesium) OR (magnesium AND dipropylacetate) OR (magnesium AND valproate AND sustained AND release AND agent) OR (magnesium AND '2 propylvalerate') | 1,136 |
| #2 | 'dementia'/exp OR dement*:ab,ti OR alzheimer*:ab,ti OR huntington*:ab,ti OR parkinson*:ab,ti OR 'lewy body':ab,ti OR 'pick disease':ab,ti OR 'cognitive impairment':ab,ti OR 'vascular dementia':ab,ti OR 'neurodegenerative disease':ab,ti OR 'neurocognitive disorder':ab,ti | 658,543 |
| #3 | #1 AND #2 | **56** |

### Cochrane Library

| | Searches | Results |
|---|---|---|
| #1 | MeSH descriptor: [Dementia] explode all trees | 6277 |
| #2 | (dement*):ti,ab,kw OR (Alzheimer*):ti,ab,kw OR (Huntington*):ti,ab,kw OR (Parkinson*):ti,ab,kw OR (Lewy body):ti,ab,kw | 31,746 |
| #3 | (Pick disease):ti,ab,kw OR (cognitive impairment):ti,ab,kw OR (vascular dementia):ti,ab,kw OR (neurodegenerative disease):ti,ab,kw OR (neurocognitive disorder):ti,ab,kw | 22,205 |
| #4 | (Valproate Magnesium) OR (Valproic acid magnesium) OR (Magnesium dipropylacetate) OR (Magnesium valproate sustained release agent) (Word variations have been searched) | 58 |
| #5 | (#1 OR #2 OR #3) AND #4 | **2** |

### EBSCO

| | Searches | Results |
|---|---|---|
| #1 | TX Valproate Magnesium OR TX Valproic acid magnesium OR TX Magnesium dipropylacetate OR TX Magnesium valproate sustained release agent OR TX Magnesium 2-propylvalerate | 214 |
| #2 | SU Dementia OR TX dement* OR TX Alzheimer* OR TX Huntington* OR TX Parkinson* OR TX Lewy body OR TX Pick disease OR TX cognitive impairment OR TX vascular dementia OR TX neurodegenerative disease OR TX neurocognitive disorder | 1,558,568 |
| #3 | #1 AND #2 | **35** |

**CNKI data**

| | Searches | Results |
|---|---|---|
| #1 | SU='痴呆'+'阿尔茨海默病'+‘阿尔茨海默症’+‘血管性痴呆’+‘额颞叶痴呆’+‘路易体痴呆’+‘帕金森痴呆’+‘神经系统退行性病变’+‘认知功能障碍’+‘认知功能下降’ | **89,303** |
| #2 | FT=‘丙戊酸镁’+‘丙戊酸镁片’+‘丙戊酸镁缓释片’ | **280** |
| #3 | #1 AND #2 | **364** |

**Wanfang data**

| | Searches | Results |
|---|---|---|
| #1 | ((丙戊酸镁) OR 丙戊酸镁片) OR 丙戊酸镁缓释片 | **792** |
| #2 | ((((主题=痴呆) OR 主题=阿尔茨海默症) OR 主题=阿尔茨海默病) OR 主题=血管性痴呆) OR 主题=额颞叶痴呆 | **172,498** |
| #3 | ((((主题=路易体痴呆) OR 主题=帕金森痴呆) OR 主题=神经系统退行性病变) OR 主题=认知功能障碍) OR 主题=认知功能下降 | **38615** |
| #4 | #2 OR #3 | **197,706** |
| #5 | #1 AND #4 | **43** |

**Supplement 2. Excluded studies after full-text review**

**1)not about dementia(n=114):**

1. 王飞, 刘晓凤, 王岩刚, 蒋小苹. 丙戊酸镁增效治疗残留型精神分裂症的对照研究. *国际精神病学杂志*. 2017;3(44).

2. 金卫东, 陈正昕, 马永春, 任志斌. 丙戊酸镁辅助治疗精神分裂症对照研究的循证医学分析. *中国药物评价*. 2012(03):225-229.

3. 胥黎明. 丙戊酸镁缓释片治疗精神分裂症冲动行为的增效作用及安全性. *当代医学*. 2020(28):179-181.

4. 黄生万, 朱秀娣. 利培酮联合丙戊酸镁缓释片治疗精神分裂症兴奋激越的效果分析. *临床医学工程*. 2016(12):1617-1618.

5. 鲍红霞. 小剂量丙戊酸镁联合抗精神病药物治疗精神分裂症患者攻击行为的临床疗效分析. *系统医学*. 2021(06):22-24.

6. 高小妹, 徐成敏, 贾春岚, 王海荣. 丙戊酸镁缓释片联合阿立哌唑对精神分裂症患者社会功能的影响. *医药导报*. 2015(10):1311-1313.

7. 马希威. 丙戊酸镁对精神分裂症的辅助治疗作用. *中国实用医药*. 2010(12):43-44.

8. 颉瑞, 王刚平, 裴根祥. 丙戊酸镁缓释片辅助治疗精神分裂症攻击行为的疗效观察. *临床精神医学杂志*. 2011(03):191-192.

9. 顾谦. 丙戊酸镁联合抗精神病药物治疗对恢复期精神分裂症患者的预后分析. *中国民康医学*. 2014(18):59+99.

10. 韩倩倩, 赫利寒. 小剂量丙戊酸镁对精神分裂症的治疗效果. *河南医学研究*. 2020(28):5299-5301.

11. 韦培钦, 谢婉婷. 奥氮平联合小剂量丙戊酸钠治疗存在攻击行为精神分裂症患者的效果. *慢性病学杂志*. 2020(08):1271-1273.

12. 陈黎明, 侯云跃. 奥氮平联合丙戊酸镁缓释片治疗精神分裂症伴兴奋冲动攻击行为对照研究. *中国实用神经疾病杂志*. 2014(13):36-38.

13. 陈维华. 抗精神病药物联合小剂量丙戊酸镁治疗精神分裂症攻击行为的比较分析. *北方药学*. 2014(05):49.

14. 陈朝晖. 小剂量丙戊酸镁联合抗精神病药物治疗精神分裂症患者攻击行为的临床效果研究. *中外医疗*. 2018(02):140-141+151.

15. 陈庭玉. 小剂量丙戊酸镁联合抗精神病药物治疗精神分裂症患者攻击行为的临床效果研究. *心理月刊*. 2020(14):116.

16. 钱昌, 陈蕴辉, 顾德君, 陈文忠. 丙戊酸镁联合治疗伴攻击行为的精神分裂症的疗效及安全性. *国际精神病学杂志*. 2018(06):1008-1010.

17. 金戈. 丙戊酸镁对精神分裂症冲动攻击行为疗效观察. *中国现代药物应用*. 2013(14):160-161.

18. 金丽娜. 奥氮平联合丙戊酸镁缓释片治疗精神分裂症伴阳性症状患者效果分析. *中国继续医学教育*. 2016(10):150-151.

19. 郭红梅，白爱萍，王军．丙戊酸镁缓释片对精神分裂症暴力攻击行为的疗效观察．*临床精神医学杂志*. 2011(02):117-118.
20. 郭春荣． 奎硫平合并丙戊酸镁缓释片治疗精神分裂症兴奋激越研究． *临床精神医学杂志*. 2007(03):183-184.
21. 邵利． 阿立哌唑联合丙戊酸镁对精神分裂症攻击行为的疗效观察． *山西医药杂志*. 2010(02):167-168.
22. 邱玲玲，王继丰．丙戊酸镁对利培酮疗效及血药浓度的影响．*辽宁医学杂志*. 2014(01):9-10.
23. 邓怀丽， 郝瑞军．喹硫平联合丙戊酸镁治疗精神分裂症攻击行为的疗效观察． *中国药物与临床*. 2020(18):3048-3050.
24. 赵顺程，白树存，董翔，廖东升．丙戊酸盐联合阿立哌唑治疗精神分裂症的疗效及对细胞免疫和体液免疫的影响．*贵州医药*. 2018(12):1483-1484.
25. 赵甫兵． 氨磺必利与阿立哌唑口崩片对精神分裂症患者阴性症状及LDL、TG、TC的影响．*现代医学与健康研究电子杂志*. 2020(02):45-47.
26. 赵燕，王在亮，吴华，公维谦．喹硫平治疗男性精神分裂症患者血药浓度与临床疗效、血清神经功能指标的相关性分析．*国际精神病学杂志*. 2017(02):219-221+225.
27. 赵丽丽， 董爱国， 朱培俊． 丙戊酸镁联合氨磺必利对精神分裂症患者神经营养因子水平及认知功能的影响．*临床医学研究与实践*. 2020(29):40-41+44.
28. 衣国河． 奥氮平联合丙戊酸镁缓释片治疗精神分裂症患者阳性症状的疗效观察． *中国医药指南*. 2019(22):60.
29. 蒋合萍， 王慢利． 奥氮平联合丙戊酸镁缓释片治疗精神分裂症患者阳性症状效果分析． *四川医学*. 2018(02):185-188.
30. 莫祥德，尹年英，胡智军，黄超．奥氮平联用丙戊酸镁缓释片治疗伴冲动攻击行为精神分裂症患者疗效分析．*临床医药文献电子杂志*. 2020(18):60-61+65.
31. 范丽伟． 丙戊酸镁缓释片联合盐酸多奈哌齐胶囊治疗老年性精神分裂症患者攻击行为的疗效观察．*山西医药杂志(下半月刊)*. 2013(03):253-255.
32. 苏海陵． 抗精神病药物联合丙戊酸镁对精神分裂症行为障碍的治疗． *辽宁医学院学报*. 2011(06):527-528.
33. 苏奎利．小剂量丙戊酸镁联合抗精神病药物治疗精神分裂症患者攻击行为的临床效果研究．*心理月刊*. 2020(13):99.
34. 苏天勋，何燕飞，张健，陈硕勋．氯氮平联合氨磺必利治疗精神分裂症疗效分析．*深圳中西医结合杂志*. 2020(08):171-173.
35. 舒卓， 周文． 氯氮平联合丙戊酸镁缓释片治疗精神分裂症攻击行为的疗效观察． *中国医药指南*. 2014(02):28-29.
36. 肖慧琼． 利培酮合并丙戊酸镁治疗精神分裂症用药现状回顾分析． *内蒙古中医药*. 2013(06):30-31.
37. 罗光琼．研究奥氮平联合丙戊酸镁缓释片治疗精神分裂症伴兴奋冲动攻击行为的临床效果．*智慧健康*. 2018(01):155-156.
38. 秦承花， 王定宏． 丙戊酸镁联合抗精神病药物对精神分裂症患者恢复期预后的影响． *深圳*

*中西医结合杂志*. 2015(20):11-12.
39. 祁晓峰. 利培酮联合丙戊酸钠治疗精神分裂症攻击行为的疗效及安全性观察. *中国处方药*. 2018(02):69-70.
40. 甘记兴, 李永瑾, 庞德兵. 小剂量丙戊酸镁联合帕利哌酮缓释片对精神分裂症患者神经营养因子水平的影响. *中华保健医学杂志*. 2021(04):366-369.
41. 王锐昊. 抗精神病药物联合小剂量丙戊酸镁治疗精神分裂症攻击行为的疗效探究. *当代医学*. 2020(13):142-143.
42. 王锋锐, 甘春连, 叶百维. 丙戊酸镁缓释片联合二甲双胍对双相情感障碍患者代谢功能及认知功能的影响. *临床心身疾病杂志*. 2021;3(27).
43. 王祖宾. 丙戊酸钠对精神分裂症行为紊乱的辅助治疗作用. *中国民康医学*. 2014(22):57-58.
44. 王海霞, 周海平. 利培酮与丙戊酸镁联合治疗对流浪精神分裂症患者认知功能的影响. *中国医院用药评价与分析*. 2016;5(16).
45. 王桂丽, 付培鑫, 李新英, 林祥吉, 纪青松, 李振江. 利培酮合并丙戊酸钠对精神分裂症患者攻击行为疗效的对照研究. *中国药物滥用防治杂志*. 2014(04):200-202.
46. 王慢利, 蒋特成. 利培酮联合丙戊酸钠治疗伴有冲动和攻击行为的精神分裂症患者的临床观察. *临床合理用药杂志*. 2013(01):72-73.
47. 王德春. 利培酮联用丙戊酸镁缓释片治疗精神分裂症患者攻击行为的疗效. *中国民康医学*. 2013(21):33-34.
48. 王小全, 马素杰, 穆小梅, 周海晓. 利培酮联合丙戊酸镁治疗伴有幻觉的精神分裂症患者的疗效观察. *中国民康医学*. 2016(10):4-6+10.
49. 王宏林. 利培酮联合丙戊酸镁对精神分裂症攻击行为的疗效观察. *中国健康心理学杂志*. 2006(04):441-442.
50. 王宏林. 丙戊酸镁联合抗精神病药治疗精神分裂症攻击行为. *临床心身疾病杂志*. 2005(01):10-11.
51. 王勇. 奥氮平合用丙戊酸镁缓释片治疗精神分裂症疗效分析. *中华全科医学*. 2013(05):730+749.
52. 王刚平, 颉瑞, 裴根祥. 喹硫平合并丙戊酸镁缓释片治疗精神分裂症攻击行为的对照研究. *精神医学杂志*. 2012(01):59-60.
53. 温钧, 李芸芳, 王刚平. 喹硫平合并丙戊酸镁缓释片治疗具有攻击行为精神分裂症的临床疗效及安全性探讨. *中国民康医学*. 2013(11):55-56.
54. 段志荣, 李惠芳. 小剂量丙戊酸镁联合抗精神病药物治疗精神分裂症患者攻击行为的临床效果研究. *中外医学研究*. 2017(16):125-126.
55. 武景霞, 林涛. 丙戊酸镁联合喹硫平治疗难治性精神分裂症的疗效研究. *实用心脑肺血管病杂志*. 2013(04):140-141.
56. 林剑峰, 邹锦山. 奥氮平联合丙戊酸镁缓释片治疗精神分裂症急性期的临床观察. *临床合理用药杂志*. 2018(31):51-52.
57. 杨实进. 小剂量丙戊酸镁联合抗精神病药物治疗精神分裂症患者攻击行为的临床疗效分析. *当代医学*. 2019(34):71-73.
58. 杨妍. 抗精神病药物联合小剂量丙戊酸镁治疗精神分裂症攻击行为的临床效果研究. *中国*

*疗养医学*. 2017(09):985-986.
59. 李红. 小剂量丙戊酸镁联合富马酸喹硫平治疗精神分裂症伴攻击行为患者的效果. *中国民康医学*. 2021(02):75-77.
60. 李玮, 穆少丽. 丙戊酸镁缓释片治疗精神分裂症攻击行为的增效作用及安全性评价. *中国药业*. 2016(13):51-53.
61. 李猛, 樊素琴. 抗精神病药物联合小剂量丙戊酸镁治疗精神分裂症攻击行为的对照研究. *中国社区医师(医学专业)*. 2011(07):41-42.
62. 李猛, 张淑芳, 贾娟, et al. 丙戊酸钠与吸烟对精神分裂症患者利培酮血药浓度和疗效的影响. *中国实用神经疾病杂志*. 2015(24):22-24.
63. 李洪兴. 评估奥氮平与丙戊酸镁缓释片对精神分裂症伴兴奋冲动攻击行为的改善. *中国医药指南*. 2019(26):118-119.
64. 李树生. 阿立哌唑联合丙戊酸镁对精神分裂症冲动控制障碍的疗效观察. *重庆医学*. 2009(07):788+798.
65. 李易俗. 碳酸锂联合喹硫平 丙戊酸镁缓释片治疗双相情感障碍躁狂发作对照研究. *临床心身疾病杂志*. 2017;5(23).
66. 李彩芳, 卫步霄. 丙戊酸镁缓释片对精神分裂症攻击行为的治疗作用. Paper presented at: 中华医学会第十一次全国精神医学学术会议、第三届亚洲神经精神药理学术会议2013; 中国北京.
67. 李彩芳, 刘元华, 王妙辉. 丙戊酸镁缓释片合用利培酮对精神分裂症冲动攻击行为的辅助治疗. *实用医技杂志*. 2013(01):69-71.
68. 李岳朋. 抗精神病药联合丙戊酸镁对伴有激越的精神分裂症患者的疗效及副作用观察. *临床医药文献电子杂志*. 2016(58):11655-11656.
69. 李冰. 小剂量丙戊酸镁联合抗精神病药物治疗精神分裂症患者攻击行为的临床效果观察. *基层医学论坛*. 2019(28):4055-4056.
70. 李丹, 徐保锋. 丙戊酸镁缓释片联合奥氮平治疗伴攻击行为的男性精神分裂症. *河南科技大学学报(医学版)*. 2017(01):58-59+65.
71. 李万顺, 于晓东, 崔宇. 奥氮平合并小剂量丙戊酸镁缓释片治疗急性期精神分裂症的对照研究. *精神医学杂志*. 2008(03):178-179.
72. 戴建磊, 欧阳泽华, 刘卫平. 丙戊酸镁缓释片联合奥氮平治疗伴冲动攻击行为精神分裂症患者的疗效和安全性研究. *中国民康医学*. 2017(05):11-12+37.
73. 徐良雄, 曾德志, 曹文韬, 朱洁琼. 联合丙戊酸镁缓释片对60例精神分裂症患者认知功能的疗效改善观察. *中国民康医学*. 2015(18):26-27.
74. 徐良雄, 刘祖松, 曾德志, 王兵华, 黄翠萍. 氯氮平联合丙戊酸镁缓释片治疗难治性精神分裂症对患者认知功能的影响. *海南医学*. 2016;11(27).
75. 徐成敏. 丙戊酸镁缓释片联合阿立哌唑对精神分裂症社会功能的影响. Paper presented at: 中华医学会第十三次全国精神医学学术会议2015; 中国山东济南.
76. 彭红波. 丙戊酸镁对精神分裂症攻击行为的疗效观察. *中国伤残医学*. 2013(07):187-188.
77. 廖凯, 汪永平, 郝光先. 阿立哌唑联合MECT对精神分裂症患者精神症状及智力水平的影响.

*贵州医药*. 2021(04):571-572.
78. 左洋. 小剂量丙戊酸镁联合喹硫平治疗精神分裂症患者的效果. *中国民康医学*. 2020(14):44-45.
79. 崔鹏, 张京华, 杨程皓, 杨会增, 韩冬昱. 丙戊酸镁缓释片对伴冲动行为的精神分裂症患者的疗效观察. *吉林医学*. 2015(16):3487-3488.
80. 孟焱, 刘文文, 陈雷音. 丙戊酸镁缓释片对精神分裂症的疗效. *河南医学研究*. 2020(28):5209-5211.
81. 孙福刚, 陈雪莲, 周海文. 小剂量丙戊酸镁联合抗精神病药物治疗精神分裂症患者攻击行为的临床效果观察. *中国现代药物应用*. 2018(24):83-84.
82. 孔荣见. 抗精神病药物联合小剂量丙戊酸镁治疗精神分裂症攻击行为的临床效果观察. *名医*. 2019(10):240.
83. 姜诚勇, 陈军良, 何国琪. 丙戊酸镁缓释片辅助治疗精神分裂症兴奋激越的效果观察. *中国药物滥用防治杂志*. 2009(03):175-177.
84. 姚丰菊, 姜峰. 利培酮联合丙戊酸镁缓释片治疗精神分裂症攻击行为的近期疗效. *职业与健康*. 2010(22):2713-2714.
85. 喻化. 奥氮平联合丙戊酸钠治疗精神分裂症伴冲动行为患者的临床观察. *当代医学*. 2016(32):151-152.
86. 周鑫, 侯媛媛, 于海. 奥氮平联合丙戊酸镁缓释片治疗精神分裂症攻击行为30例. *中国药业*. 2015(07):87-88.
87. 周继. 小剂量丙戊酸镁联合氯氮平治疗精神分裂症的临床疗效及其安全性. *临床合理用药杂志*. 2021(18):124-126.
88. 周益辉, 钟跃峰, 曾德志, 戴列军. 丙戊酸镁缓释片联合齐拉西酮治疗精神分裂症攻击行为的疗效观察. Paper presented at: 中华医学会第十次全国精神医学学术会议2012; 中国江苏南京.
89. 周海平, 王海, 王海霞, 钟远惠. 利培酮口服液合并丙戊酸镁治疗精神分裂症兴奋激越的疗效与安全性研究. *中华临床医师杂志(电子版)*. 2016(07):930-933.
90. 周升宝, 孙晓丹, 田月礼, 秦巍, 胡冰. 丙戊酸镁缓释片对精神分裂症攻击行为治疗的增效作用及安全性. *中国健康心理学杂志*. 2015(04):484-487.
91. 周为, 谢维爵, 胡耀之. 奥氮平合并丙戊酸镁缓释片治疗伴攻击行为的男性精神分裂症临床观察. *临床精神医学杂志*. 2014(03):194-196.
92. 吴秀萍, 杨庆华, 张丽梅, 周淑琼. 奥氮平联合丙戊酸镁缓释片治疗精神分裂症伴阳性症状患者的对照研究. *西部医学*. 2013(12):1842-1844.
93. 吴忠海, 王洪娟. 奥氮平联合丙戊酸镁缓释片对伴攻击行为精神分裂症患者的疗效研究. *继续医学教育*. 2015(06):102-104.
94. 吴兴曲, 贾婷, 禹晓东, 杨来启, 黄旭光, 马文涛. 氯氮平联合阿立哌唑治疗难治性精神分裂症的临床效果及对糖脂代谢和胰岛素抵抗的影响. *解放军医药杂志*. 2019(10):71-75.
95. 史月仙, 戴天刚, 易鹏程. 奥氮平合并丙戊酸镁缓释片治疗精神分裂症急性期兴奋激越研究. *现代实用医学*. 2010(04):398-399.
96. 卢智慧. 阿立哌唑合并丙戊酸镁缓释片治疗女性精神分裂症患者的对照研究. *医疗装备*. 2016(12):140.

97. 刘杰. 丙戊酸镁对精神分裂症兴奋激越行为的辅助疗效观察. *社区医学杂志*. 2012(14):31-32.
98. 刘忠, 王立娟, 孙玉涛. 丙戊酸镁缓释片对精神分裂症兴奋状态的疗效观察. *中国健康心理学杂志*. 2012(11):1630-1631.
99. 刘忠, 李和军, 裴双义. 丙戊酸镁合并利培酮治疗精神分裂症血药浓度变化及临床疗效. *中国健康心理学杂志*. 2014(08):1141-1143.
100. 刘忠, 孙玉涛, 王立娟. 丙戊酸镁联合帕利哌酮治疗精神分裂症23例疗效观察. *山东医药*. 2011(23):54-55.
101. 刘夫娟. 小剂量丙戊酸镁增效联合心理干预治疗对精神分裂症伴阳性症状患者的影响. *心理月刊*. 2021(06):82-83.
102. 冯莉, 杨静, 郭年春, 高艳山. 小剂量丙戊酸镁联合抗精神病药物治疗精神分裂症患者攻击行为的临床价值研究. *人人健康*. 2020(14):669.
103. 何晓华. 抗精神病药物联合小剂量丙戊酸镁对精神分裂症患者生活质量的影响. *吉林医学*. 2012(04):751-752.
104. 何新年. 抗精神病药物联合小剂量丙戊酸镁治疗精神分裂症攻击行为的效果分析. *临床医药文献电子杂志*. 2020(30):168.
105. 伏彩霞, 刘烨, 唐娅娟, 祁鑫川. 喹硫平联合丙戊酸镁缓释片治疗精神分裂症攻击行为的疗效观察. Paper presented at: 国际数字医学会数字中医药分会成立大会暨首届数字中医药学术交流会 2016; 中国广东珠海.
106. 伏彩霞, 刘烨, 唐娅娟, 祁鑫川. 喹硫平联合丙戊酸镁缓释片治疗精神分裂症攻击行为的疗效观察. Paper presented at: 中华医学会第十三次全国精神医学学术会议2015; 中国山东济南.
107. 付永强. 利培酮口服液合并丙戊酸镁治疗精神分裂症兴奋激越的疗效分析. *实用中西医结合临床*. 2016(09):72-73.
108. 尹延明, 赵学利, 周桦, 宋存衡. 抗精神病药物联合小剂量丙戊酸镁在精神分裂症患者中的疗效观察及对BDNF、GDNF水平的影响研究. *国际精神病学杂志*. 2019(01):96-98+102.
109. 万春平. 抗精神病药物联合小剂量丙戊酸镁治疗精神分裂症攻击行为的对照研究. *齐齐哈尔医学院学报*. 2016(10):1324-1325.
110. Ristić AJ, Vojvodić N, Janković S, Sindelić A, Sokić D. The frequency of reversible parkinsonism and cognitive decline associated with valproate treatment: a study of 364 patients with different types of epilepsy. *Epilepsia.* 2006;47(12):2183-2185.
111. Leclair-Visonneau L, Rouaud T, Debilly B, et al. Randomized placebo-controlled trial of sodium valproate in progressive supranuclear palsy. *Clin Neurol Neurosurg.* 2016;146:35-39.
112. Ibrahim I, Tobar S, Fathi W, et al. Randomized controlled trial of adjunctive Valproate for cognitive remediation in early course schizophrenia. *J Psychiatr Res.* 2019;118:66-72.
113. Haghighi M, Bajoghli H, Bigdelou G, Jahangard L, Holsboer-Trachsler E, Brand S. Assessment of cognitive impairments and seizure characteristics in electroconvulsive therapy with and without sodium valproate in manic patients. *Neuropsychobiology.* 2013;67(1):14-24.
114. Aldenkamp AP, Baker G, Mulder OG, et al. A multicenter, randomized clinical study to evaluate the effect on cognitive function of topiramate compared with valproate as add-on therapy to carbamazepine in patients with partial-onset seizures. *Epilepsia.* 2000;41(9):1167-1178.

**2)not about oral VPM(n=73):**

陈业鹏. 美金刚联合盐酸多奈哌齐治疗阿尔茨海默病伴精神行为症状的临床效果观察. 中国医药科学. 2020(10):60-62.
2. 胡春丽. 多奈哌齐联合奥氮平治疗老年痴呆合并精神行为障碍患者的效果. 中国民康医学. 2020(05):100-102.
3. 梁东旭. 多奈哌齐联合奥氮平治疗老年痴呆伴发精神行为症状患者的临床效果. 中国当代医药. 2020(31):76-78+82.
4. 陈立勇, 刘秀丽. 丙戊酸钠缓释片对阿尔茨海默病患者精神行为症状的疗效及安全性. 齐齐哈尔医学院学报. 2017(03):255-257.
5. 戴晓燕. 喹硫平联合丙戊酸钠缓释片治疗阿尔茨海默病精神行为症状的疗效观察. 新疆医科大学学报. 2019(02):203-206.
6. Vance H, Kauffman C, Miller B, Mansour T. Treatment of agitation using Depakote: a patient with dementia. Psychiatry. 2003;66(4):358-359.
7. Tariot PN, Raman R, Jakimovich L, et al. Divalproex sodium in nursing home residents with possible or probable Alzheimer Disease complicated by agitation: a randomized, controlled trial. Am J Geriatr Psychiatry. 2005;13(11):942-949.
8. Sival RC, Haffmans PM, van Gent PP, van Nieuwkerk JF. The effects of sodium valproate on disturbed behavior in dementia. J Am Geriatr Soc. 1994;42(8):906-907.
9. Sival RC, Haffmans PM, Jansen PA, Duursma SA, Eikelenboom P. Sodium valproate in the treatment of aggressive behavior in patients with dementia--a randomized placebo controlled clinical trial. Int J Geriatr Psychiatry. 2002;17(6):579-585.
10. Sival RC, Duivenvoorden HJ, Jansen PA, Haffmans PM, Duursma SA, Eikelenboom P. Sodium valproate in aggressive behaviour in dementia: a twelve-week open label follow-up study. Int J Geriatr Psychiatry. 2004;19(4):305-312.
11. Profenno LA, Jakimovich L, Holt CJ, Porsteinsson A, Tariot PN. A randomized, double-blind, placebo-controlled pilot trial of safety and tolerability of two doses of divalproex sodium in outpatients with probable Alzheimer's disease. Curr Alzheimer Res. 2005;2(5):553-558.
12. Porsteinsson AP, Tariot PN, Jakimovich LJ, et al. Valproate therapy for agitation in dementia: open-label extension of a double-blind trial. Am J Geriatr Psychiatry. 2003;11(4):434-440.
13. Porsteinsson AP, Tariot PN, Erb R, Gaile S. An open trial of valproate for agitation in geriatric neuropsychiatric disorders. Am J Geriatr Psychiatry. 1997;5(4):344-351.
14. Porsteinsson AP, Tariot PN, Erb R, et al. Placebo-controlled study of divalproex sodium for agitation in dementia. Am J Geriatr Psychiatry. 2001;9(1):58-66.
15. Nutt J, Williams A, Plotkin C, Eng N, Ziegler M, Calne DB. Treatment of Parkinson's disease with sodium valproate: clinical, pharmacological, and biochemical observations. Can J Neurol Sci. 1979;6(3):337-343.
16. Narayan M, Nelson JC. Treatment of dementia with behavioral disturbance using divalproex or a combination of divalproex and a neuroleptic. J Clin Psychiatry. 1997;58(8):351-354.
17. Mellow AM, Solano-Lopez C, Davis S. Sodium valproate in the treatment of behavioral disturbance in dementia. J Geriatr Psychiatry Neurol. 1993;6(4):205-209.
18. Meinhold JM, Blake LM, Mini LJ, Welge JA, Schwiers M, Hughes A. Effect of divalproex sodium on behavioural and cognitive problems in elderly dementia. Drugs Aging. 2005;22(7):615-626.
19. Lott AD, McElroy SL, Keys MA. Valproate in the treatment of behavioral agitation in elderly patients with dementia. J Neuropsychiatry Clin Neurosci. 1995;7(3):314-319.
20. Kunik ME, Puryear L, Orengo CA, Molinari V, Workman RH, Jr. The efficacy and tolerability of divalproex sodium in elderly demented patients with behavioral disturbances. Int J Geriatr Psychiatry. 1998;13(1):29-34.
21. Herrmann N. Valproic acid treatment of agitation in dementia. Can J Psychiatry. 1998;43(1):69-72.
22. Fleisher AS, Truran D, Mai JT, et al. Chronic divalproex sodium use and brain atrophy in Alzheimer disease. Neurology. 2011;77(13):1263-1271.
23. Herrmann N, Lanctôt KL, Rothenburg LS, Eryavec G. A placebo-controlled trial of valproate for agitation and aggression in Alzheimer's disease. Dement Geriatr Cogn Disord. 2007;23(2):116-119.

24. Forester B, Vanelli M, Hyde J, et al. Report on an open-label prospective study of divalproex sodium for the behavioral and psychological symptoms of dementia as monotherapy and in combination with second-generation antipsychotic medication. Am J Geriatr Pharmacother. 2007;5(3):209-217.
25. 齐锞, 郭长娥, 董子涵. 奥氮平治疗老年痴呆患者的临床疗效及对精神行为症状的影响. 首都食品与医药. 2019;16(26).
26. 黄建申, 沈英生, 黄金武. 银杏叶提取物对阿尔茨海默病患者认知功能的效果及其对相关因子的影响. 世界中医药. 2016(12):2630-2633.
27. 魏悯. 多奈哌齐联合美金刚对阿尔茨海默病患者认知功能与行为能力的作用及安全性. 武汉大学学报(医学版). 2016(03):461-463+511.
28. 马莉, 赵敏强, 范思海, et al. 肠道益生菌联合加兰他敏片对阿尔茨海默病患者认知功能及治疗安全性的影响研究. 中国药物与临床. 2021(14):2427-2429.
29. 马楠. 奥氮平治疗对改善老年痴呆精神行为障碍的临床效果研究. 当代医学. 2019(01):32-34.
30. 马建法. 银杏内酯注射液联合多奈哌齐对老年痴呆患者的影响. 实用中西医结合临床. 2020(17):52-53.
31. 马学森, 李凌, 曾珍. 多奈哌齐对阿尔茨海默病并精神异常患者认知功能及日常生活自理能力的影响. 中国老年学杂志. 2015(19):5579-5580.
32. 陈礼军, 吴晓娟. 醒脑益智汤联合盐酸多奈哌齐治疗老年性痴呆临床研究. 新中医. 2021(02):49-52.
33. 陈晓华, 刘博, 曹娜. 鼠神经生长因子联合多奈哌齐治疗老年痴呆的效果观察. 实用临床医药杂志. 2019(15):24-26.
34. 陈少光. 氨磺必利联合奥氮平对精神分裂症患者糖脂代谢指标及认知功能的影响. 实用中西医结合临床. 2021(13):60-61+105.
35. 赵莹. 盐酸多奈哌齐治疗老年痴呆患者对其智力及生活能力影响观察. 健康之路. 2017(04):122-123.
36. 赵玲洁, 刘华瑞. 富马酸喹硫平联合奥氮平对阿尔茨海默病伴精神行为障碍的疗效及安全性分析. 甘肃医药. 2021(01):26-28.
37. 谢渭根, 张烈, 陶涛. 盐酸美金刚治疗阿尔茨海默病伴精神行为症状效果观察. 中国乡村医药. 2017(17):5-6.
38. 董豹. 喹硫平与氟哌啶醇治疗阿尔茨海默病患者精神行为症状临床效果研究. 中国现代药物应用. 2014(17):144-146.
39. 董永华. 石杉碱甲片对阿尔茨海默病患者的应用效果. 河南医学研究. 2020(27):5116-5118.
40. 茹文学. 益智聪脑颗粒联合多奈哌齐片治疗老年痴呆效果观察. 慢性病学杂志. 2019(03):467-468.
41. 范莹, 刘辉. 盐酸多奈哌齐治疗阿尔茨海默病的效果. 临床医学. 2019(09):89-91.
42. 糜屈, 第五永长. 盐酸美金刚联合多奈哌齐治疗中、重度阿尔茨海默病的临床研究. 中国现代医生. 2014(18):42-44+47.
43. 申志强. 氯氮平与齐拉西酮对老年痴呆患者认知功能的影响. 慢性病学杂志. 2021(02):288-289+292.
44. 王雪莲. 奥氮平和阿立哌唑用于老年痴呆患者精神行为症状治疗的临床疗效. 影像研究与

医学应用. 2017(09):12-14.
45. 王绍利. 多重感官刺激对阿尔茨海默病患者认知水平及精神行为症状的影响. 护理实践与研究. 2021(02):287-289.
46. 王博, 耿英华. 氟西汀联合喹硫平治疗伴有强迫症状精神分裂症患者的临床效果观察. 中国药物滥用防治杂志. 2021(01):67-70+76.
47. 王健. 马来酸咪达唑仑片联合富马酸喹硫平治疗老年痴呆并发睡眠障碍临床观察. 中西医结合心脑血管病杂志. 2017(07):861-863.
48. 熊江红. 用五氟利多治疗老年性痴呆的效果研究. 当代医药论丛. 2016(20):120-121.
49. 梁献升, 黄光位, 林惠贤. 多奈哌齐治疗老年痴呆症患者有效性分析. 中国医药科学. 2020(05):69-71.
50. 杨学东. 奥拉西坦在老年性痴呆治疗中的疗效探讨. 中外医疗. 2015(29):124-125.
51. 李瑞天, 赵园园, 蔡灵钰, 吴玉泉. 盐酸多奈哌齐对阿尔茨海默病患者认知功能及氧化应激水平的影响. 中国医药. 2020(03):382-385.
52. 李海旺. 舒血宁联合脑蛋白水解物治疗老年痴呆的临床效果研究. 中国农村卫生. 2017(24):17.
53. 朱红星. 奥氮平治疗阿尔茨海默病的效果及安全性评价. 中国医学创新. 2015(19):137-138.
54. 朱佳, 陈菲, 美丽班娜, et al. 普罗布考联合多奈哌齐治疗阿尔茨海默病的疗效. 中国继续医学教育. 2021(09):165-168.
55. 曹业才. 舒必利联合阿立哌唑治疗难治性精神分裂症的临床疗效分析. 中国医药指南. 2021(11):90-91.
56. 徐哲, 陈浙丽. 喹硫平联合心境稳定剂治疗老年阿尔茨海默病精神行为症状的临床观察. 健康研究. 2019(02):185-188.
57. 张惠莉, 陈国军, 王淑梅, 郑文权. 盐酸多奈哌齐联合石杉碱甲对老年血管性痴呆患者认知功能和运动功能的影响. 临床研究. 2021(07):107-108.
58. 张建强, 高红安, 宋程光, 曹群, 李莉. 酒石酸卡巴拉汀联合盐酸多奈哌齐对阿尔茨海默病病人轻度认知功能障碍的影响. 中西医结合心脑血管病杂志. 2017(03):368-370.
59. 张宗义. 盐酸多奈哌齐对老年痴呆症患者生活能力、精神状态的影响. 中外医疗. 2021(20):90-93.
60. 安庆文. 尼膜同与盐酸多奈哌齐治疗老年性痴呆症的疗效比较. 中国实用神经疾病杂志. 2016(08):127-128.
61. 孙辉. 氟哌啶醇联合利培酮治疗痴呆患者精神行为症状的疗效观察. 实用临床医药杂志. 2015(11):121-122.
62. 孙乃建, 李天龙, 钟云川, 宋亚玲. 氟哌啶醇与利培酮治疗痴呆患者精神行为症状的疗效及安全性比较研究. 中外医疗. 2014(08):103-104.
63. 姜诚勇, 谢谓根, 陈军良, 田利萍. 卡巴拉汀联合阿托伐他汀对阿尔茨海默病患者脑内促炎症细胞因子的影响. 中国医师杂志. 2021(04):617-619.
64. 吕云峰. 盐酸多奈哌齐联合用药方案延缓老年痴呆患者病情的临床分析. 中国实用医药. 2018(02):107-109.
65. 华浩水, 邵剑锋, 陆源昕, 廖峥娈. 小剂量齐拉西酮联合盐酸多奈哌齐治疗老年痴呆的疗效

及对患者糖脂代谢的影响. 中华全科医学. 2019(11):1860-1863.
66. 何金珠. 奥氮平治疗老年痴呆伴精神行为障碍分析. 中国卫生标准管理. 2020(03):75-77.
67. Zádori D, Geisz A, Vámos E, Vécsei L, Klivényi P. Valproate ameliorates the survival and the motor performance in a transgenic mouse model of Huntington's disease. Pharmacol Biochem Behav. 2009;94(1):148-153.
68. Yeh HL, Tsai SJ. Lithium may be useful in the prevention of Alzheimer's disease in individuals at risk of presenile familial Alzheimer's disease. Med Hypotheses. 2008;71(6):948-951.
69. Tariot PN. Clinical trials of amyloid-based therapies for Alzheimer's disease. CNS Spectr. 2007;12(1 Suppl 1):7-10.
70. Porsteinsson AP. Divalproex sodium for the treatment of behavioural problems associated with dementia in the elderly. Drugs Aging. 2006;23(11):877-886.
71. Mizukami K, Hatanaka K, Ishii T, et al. Effects of sodium valproate on behavioral disturbances in elderly outpatients with dementia. Geriatr Gerontol Int. 2010;10(4):324-326.
72. Kumamoto A, Chiba Y, Suda A, Hishimoto A, Kase A. A Severe Dementia Case in End of Life Care with Psychiatric Symptoms Treated by Perampanel. J Epilepsy Res. 2021;11(1):93-95.
73. Dolder C, McKinsey J. Low-dose divalproex in agitated patients with Alzheimer's disease. J Psychiatr Pract. 2010;16(1):63-67.

## 3)not original article(n=3):

1. Liu J, Wang LN. Efficacy and safety of valproic acid in dementia: A systematic review with meta-analysis. Arch Gerontol Geriatr. 2020;89:104091.
2. Xiao H, Su Y, Cao X, Sun S, Liang Z. A meta-analysis of mood stabilizers for Alzheimer's disease. J Huazhong Univ Sci Technolog Med Sci. 2010;30(5):652-658.
3. Lonergan E, Luxenberg J. Valproate preparations for agitation in dementia. Cochrane Database Syst Rev. 2009(3):Cd003945.

## 4)not clinical study(n=3):

1. Chiu CT, Liu G, Leeds P, Chuang DM. Combined treatment with the mood stabilizers lithium and valproate produces multiple beneficial effects in transgenic mouse models of Huntington's disease. Neuropsychopharmacology. 2011;36(12):2406-2421.
2. Liu CH, Liao WC, Li HH, et al. Treatment with the combination of clavulanic acid and valproic acid led to recovery of neuronal and behavioral deficits in an epilepsy rat model. Fundam Clin Pharmacol. 2021.
3. Li YZ, Liu YJ, Zhang W, Luo SF, Zhou X, He GQ. Combined treatment with valproic acid and estrogen has neuroprotective effects in ovariectomized mice with Alzheimer's disease. Neural Regen Res. 2021;16(10):2078-2085.

## 5)not report diagnostic criteria of dementia(n=3):

1. 高海燕. 多药联合治疗老年痴呆精神行为障碍的疗效及不良反应研究. 中西医结合心血管病电子杂志. 2017;14(5).
2. 林彩云, 蔡齐健, 卢仪. 丙戊酸镁缓释片联合奥氮平治疗阿尔茨海默病患者的临床效果. 中国当代医药. 2018;13(25).
3. 史丽娜. 丙戊酸镁缓释片联合盐酸多奈哌齐胶囊治疗老年痴呆症对患者精神行为的影响. 当代医药论丛. 2020;17(18).

## 6)the outcome data unclear(n=1):

1. 郑左红, 宋肖潇. 丙戊酸镁联合多奈哌齐对老年痴呆认知行为的影响. *医学信息*. 2019;z1(32).

**7)VPM monotherapy(n=3):**

1. 赵红梅, 徐月勤. 奥氮平与丙戊酸镁缓释片对老年痴呆患者的干预效果对比. 中国处方药. 2018;6(16).
2. 李宁, 李梅香, 张冬红, 王丽娜. 老年期痴呆患者激越行为的药物控制:丙戊酸镁与利培酮分组治疗对照. 中国临床康复. 2006;30(10).
3. Li N, Li MX, Zhang DH, Wang LN. Drug control for agitation behavior of patients with gerontic dementia: Magnesium valproate versus risperidone in grouping treatment. Chinese Journal of Clinical Rehabilitation. 2006;10(30):158-159.

**8)not report outcome of interest(n=1):**

1. 张飞. 丙戊酸镁联合多奈哌齐对老年痴呆认知行为的改善研究. 医学食疗与健康. 2021;8(19).

**9)use duplicate data(n=3):**

1. 范丽伟. 丙戊酸镁缓释片联合盐酸多奈哌齐胶囊改善老年性痴呆患者认知行为的疗效观察. 中国老年学杂志. 2014;2(34).
2. 谢林珠, 王加磊. 合并使用丙戊酸镁缓释片治疗阿尔茨海默病精神行为症状的疗效观察. Paper presented at: 中华医学会第十次全国精神医学学术会议2012; 中国江苏南京.
3. 谢林珠. 合并使用丙戊酸镁缓释片治疗阿尔茨海默病精神行为症状的疗效观察. 2012.
